# Investigation of the Environmental and Socio-Economic Characteristics of Counties with a High Asthma Burden to Focus Asthma Action in Utah

**DOI:** 10.3390/ijerph17145251

**Published:** 2020-07-21

**Authors:** Maureen Vowles, Ruth Kerry, Ben Ingram, Linda Mason

**Affiliations:** 1 Department of Public Health and Policy, University of Liverpool, Liverpool L69 3GB, UK; 2 Department of Geography, Brigham Young University, Provo, UT 84602, USA; ruth_kerry@byu.edu; 3 Faculty of Engineering, Universidad de Talca, Curicó 3460000, Chile; ingrambr@gmail.com; 4 Liverpool School of Tropical Medicine, Liverpool L3 5QA, UK; Linda.Mason@lstmed.ac.uk

**Keywords:** air pollution, Utah, asthma, elevation, altitude, aridity, PM_2.5_, fine particulate matter, desert dust, inversion, mine dust, smoking, adult asthma prevalence, asthma emergency room visits, public health

## Abstract

Rising adult asthma prevalence (AAP) rates and asthma emergency room (AER) visits constitute a large burden on public health in Utah (UT), a high-altitude state in the Great Basin Desert, USA. This warrants an investigation of the characteristics of the counties with the highest asthma burden within UT to improve allocation of health resources and for planning. The relations between several predictor environmental, health behavior and socio-economic variables and two health outcome variables, AAP and AER visits, were investigated for UT’s 29 counties. Non-parametric statistical comparison tests, correlation and linear regression analysis were used to determine the factors significantly associated with AER visits and AAP. Regression kriging with Utah small area data (USAD) as well as socio-economic and pollution data enabled local Moran’s I cluster analysis and the investigation of moving correlations between health outcomes and risk factors. Results showed the importance of desert/mining dust and socio-economic status as AAP and AER visits were greatest in the south of the state, highlighting a marked north–south divide in terms of these factors within the state. USAD investigations also showed marked differences in pollution and socio-economic status associated with AAP within the most populous northern counties. Policies and interventions need to address socio-economic inequalities within counties and between the north and south of the state. Fine (PM_2.5_) and coarse (PM_10_) particulate matter monitors should be installed in towns in central and southern UT to monitor air quality as these are sparse, but in the summer, air quality can be worse here. Further research into spatiotemporal variation in air quality within UT is needed to inform public health interventions such as expanding clean fuel programs and targeted land-use policies. Efforts are also needed to examine barriers to routine asthma care.

## 1. Introduction

Rising adult asthma prevalence (AAP) rates and asthma emergency room (AER) visits constitute a large burden on public health in Utah (UT) and there is considerable variation within the state [1]. This warrants an investigation of the characteristics of the counties with the highest asthma burden within UT to improve allocation of limited health resources and for planning.

According to the 2014 National Health Interview Survey (NHIS), 17.7 million or 7.4% of US adults reported current doctor-diagnosed asthma [2]. Additionally, asthma accounted for 1.8 million visits to emergency rooms in 2011 [3] and 372,000 hospital admissions in 2012 [4]; with estimated annual costs of USD 56 billion in the United States [5]. However, while asthma mortality rates have been declining [6], prevalence rates are rising [7]. Utah is no exception to this with AAP rates rising by 28.6% from 7 to 9% in the period 2001–2015 [1,8]. However, there are wide regional variations in prevalence rates within UT.

Geographical variations in asthma prevalence reflect differences in health behaviors, environmental factors and socio-economic differences. AAP rates provide an appropriate indicator to track the disease burden [1], and smoothed county-level AER visit rates can be used as a measure of asthma exacerbation. Costs associated with AER visits climbed from USD 2.4 to over USD 7 million between 2000 and 2010 in UT alone [1]. Such increases led to the development of federal public health (PH) initiatives like the Healthy People 2020 (HP2020) Objective 3 target goal for Respiratory Diseases (RD-3) to “reduce hospital emergency department visits for asthma” [1] (p. 7). Based on 2015 annual data of 55.0 visits per 10,000 population, HP2020 targets of 49.5 were not met overall, although goals were met in females (47.2) but not attained in males (63.2) [1,2].

Although asthma has a strong underlying genetic component, it is often referred to as an environmental disease as symptoms are commonly exacerbated by dust, allergens, tobacco smoke and atmospheric pollution [9].

With approximately three million deaths worldwide directly ascribable to air pollution, the 2016 World Health Organization (WHO) report [10] declared outdoor air pollution a global health crisis and created strategic priorities [11]. More recent longitudinal studies suggest that the WHO global mortality figure may be significantly underestimated [12].

The Environmental Protection Agency (EPA) monitors six main classes of air pollutants—particulate matter, sulfur- and nitrogen- oxides, carbon monoxide, ozone and lead [13] which primarily originate from industry, agriculture and traffic. Fine particulate matter, particles less than 2.5 micrometers (PM_2.5_), lodging in the respiratory tract have been associated with asthma and other negative respiratory and extra-respiratory health outcomes [13], and air quality improvements have been linked with reductions in asthma prevalence, mortality and morbidity [14,15]. Of the six key pollutants monitored by the EPA, PM_2.5_ provides the best proxy indicator of ambient air pollution for health effects [10].

Reductions in lung volume, related to increased carbon monoxide from less-efficient burning of fossil fuels at lower atmospheric pressure, and intensifying PM dosage at high elevations coupled with desert dust, pose special challenges for residents of UT—a high-altitude state in the Great Basin Desert, USA. In addition, occupational and general exposure to dust rich in potentially toxic compounds from mining activities in rural UT could be a problem [16]. Northern UT valleys experience winter temperature inversions where pollutants get trapped with cold air in the valleys by warm air above [17]. Two UT urban areas: Salt Lake City-Provo-Orem and Logan, ranked 6th and 7th, respectively, nationally in the top ten worst areas for short-term PM pollution in the 2016 State of the Air Report [18]. In the 2019 Report [19], Salt Lake City still ranked 8th, while Logan dropped to 11th nationally. Additionally, red air days where daily PM_2.5_ levels exceed National Ambient Air Quality Standards (NAAQS) of 35 μg m^3^ have been observed in UT. Whiteman et al. [20] noted 18 “red air days” in the Salt Lake Valley, UT; the nation’s worst prolonged pollution inversion event with PM_2.5_ exceeding 130 μg/m^3^ was in UT’s Cache Valley in January 2004 [21]. Rises in AAP rates in UT [1] make investigations of links with air pollution an important public health issue.

The 1970 Clean Air Act (CAA, 1970: Title 42, Chapter 85) set the stage for public health protection, contemporary air quality standards and stringent PM monitoring [22]. The EPA revised PM_2.5_ standards in 2012 by lowering the allowable annual average concentrations from 15 to 12 μg m^3^ [23], although 2016 WHO guidelines [10] recommend annual limits of 10 μg m^3^. However, a large 2017 study of deaths from in USA from 2000 to 2012 [24], showed a significant association between short-term PM_2.5_ exposures at levels well below current NAAQS and increased mortality in elderly patients (>65 years). In 2011, 11 and 17 of the 29 UT counties did not meet NAAQS for PM_2.5_ annual daily average and daily upper limits, respectively [25]. Individual states monitor PM_2.5_ levels, but current EPA regulations are not state-specific; are defined as an annual daily average; and do not reflect dosage/intensity nor altitude/topography; and adjustments to PM_2.5_ air quality standards are not made in high-altitude urban areas [26]. Furthermore, Johnson (2011) noted that within UT the high asthma burden could be associated with long-term exposure to mining and desert dust which tends to have a larger particle size [27]. As dust storms are periodic events, rather than constant like urban traffic pollution, their effects have not been routinely monitored, however, dust storm air quality can be far worse than during temperature inversions [27]. Indeed, Goodman et al. (2019) note that 90% of northern UT’s dust comes from shrinking lakes to the west and has a larger particle size (PM_10_) [28].

This study aims to identify characteristics of populations and their environments that have high AAP and AER visits in UT by studying geographical factors relevant to the high-desert environment, and considering the diverse sources of air pollution which include crustal silicates (dust) and polycyclic aromatic hydrocarbons from fossil fuel combustion, tobacco smoke [29] and mine dust.

### 1.1. Conceptual Model

A conceptual model identifying the anticipated links with environmental factors and justifying the choice of variables used in this study (Table 1) follows. Three types of factor linked to asthma are investigated here: environmental, behavioral and socio-economic.

#### 1.1.1. Environmental Factors

A literature review of 1130 epidemiological studies from 2011 [29] identified urban air pollution, desert dust and tobacco smoke as the main risk factors for asthma.

Positive associations were found between asthma symptoms and PM_2.5_ in large US ecological studies [30,31,32]. Additionally, rates of asthma exacerbation due to increases in PM_2.5_ levels were found in a large geospatial study [33]. A UT study of AER visits [34] found weak positive associations between asthma exacerbation and short-term PM pollution during winter inversions. Two other USA studies [35,36] also found links between PM_2.5_ levels from winter inversions and traffic pollution, and lower respiratory disease. Given these findings, asthma exacerbation during red air days [20] is likely.

A time-series study of AER visits in Kuwait [37] found increases in AER visits on days during dust storms. However, no significant association was found between asthma exacerbation and dust events in Japan [38]. Conversely, two studies [39,40] found positive associations between relative humidity and AER visits.

Austrian longitudinal studies [41] showed that living at high altitude presents a greater pediatric risk of hospitalization for asthma. However, it has been argued that high altitude may be beneficial for well-controlled acclimatized asthmatic patients [42]. A 2017 study from Mexico [43] also suggested a protective effect of high altitude with significantly reduced risk of new asthma onset at altitudes above 1500 m. No studies of this nature have been conducted in UT to date.

Occupational and general exposure to mine and other dust has been associated with elevated asthma rates in previous studies [44,45,46]. Mining plays a major role in the economy of UT, with UT ranking 3rd nationally in the value of non-fuel minerals produced in 2009 and accounting for about 7% of the total US nonfuel mineral production [16]. Additionally, 114 large mines (including coal mines) and 195 small mines were listed as active in UT in 2009 [16]. While temporal studies have shown an increase in AER visits during dust storms [37], there is little county-level information about dust storm frequency and the mobilization of dust from mining tailings. However, Dunway et al. [47] presented maps of the Western USA of areas with a high risk of wind erosion. These areas were locations with an aridity index (AI) of <0.2 and where fine sand-sized particles dominate. There are large areas with these characteristics in south eastern UT as well as about one third of the state of Nevada which is directly to the west of UT [47] and UT’s prevailing winds come from the west [28]. Goodman et al. [28] note that 90% of the dust in Northern UT comes from dried lake beds to the west, but that dust from mines is rich in toxins such as copper and antimony.

Based on the findings mentioned, the environmental variables included in this study (Table 1) are: PM_2.5_, red air days, aridity index (AI), elevation, estimated mine area, total mines and wind erosion risk.

#### 1.1.2. Behavioral Health Factors

A statistically significant association between active smoking and AER visits was found in a large US survey of asthmatic adults [48] and another large study [49] controlling for the “healthy smoker effect” had similar findings. A 2010 United States Centers for Disease Control and Prevention (CDC) study found a significant association between obesity, asthma development, and poor control of symptoms [50]. Another study demonstrated increasing asthma severity with obesity in American adults [51]. Based on the findings of the studies mentioned, the behavioral health variables included in this study (Table 1) are: % smoking and % obesity.

#### 1.1.3. Socio-Economic Factors

A US study of asthma prevalence using almost one million data from 2000–2003, showed that western states experienced the highest increases in asthma prevalence in rural areas [52] which was partly linked to lower access to health providers in rural areas impacting disease management. UT, however, did not follow this pattern and had the largest increases in urban areas, suggesting the role of human activity from vehicular/household emissions and winter inversions exacerbating asthma rates [53].

The rural–urban divide in the American west and UT has also been linked to a number of social determinants of health and deprivation [54,55]. Indeed, nationally, and for each individual state, AAP is greatest for the two lowest income bands (<USD 15,000 and USD 15,000–25,000) [56], suggesting that poor housing, diet, manual labor and lack of access to healthcare all influence AAP. A study in Texas [57] showed uninsured adults had significantly higher rates of hospitalization than insured individuals following dust and wind events. Given the dusty desert environment and winter inversions, these findings have important implications for UT where 2012 county-level rates of uninsured ranged from 13% in Davis and Morgan Counties to 28% in San Juan County [25]. A large Korean study [58] showed significantly higher AER visit rates among un- and under-insured individuals. The health improvement index (HII) developed by the UT Department of Health [55] is a composite measure of social determinants of health by geographic area based on nine socio-economic indicators. This index could be used to assess the general impact of socio-economic status on AAP and AER visits. UT is divided into 99 small areas based on ZIP codes, local health district, county boundaries, and communication with local communities. First defined in 1997, these Utah small areas data (USAD) spatial units will be used throughout this study.

Racial links with asthma prevalence have been investigated to determine if there are racial genetic pre-dispositions to asthma [59]. Nationally within the USA, Native American and Black racial groups had the largest AAP in 2016–2018 with rates of approximately 10.5% and 9.6%, respectively, compared with 8.2% for white non-Hispanics [2]. However, it is not known if high rates in these groups are related to genetic pre-disposition or economic disadvantage [60,61]. UT does not have a large black population, but the native American population is large in some rural counties that have reservations. Differences in rates of insured individuals by race could also influence the rate of AER visits.

Based on previous findings, the socio-economic variables included in this study (Table 1) are: HII, % native American, % uninsured, % poverty, median household income (MHHI), % unemployment and population density.

## 2. Materials and Methods

### 2.1. Study Design

The descriptive ecological study design used here is appropriate for making geographical comparisons and investigating associations between asthma outcome variables and several factors at the population level [62]. However, population-level associations do not necessarily hold true at the individual level and the study cannot determine cause and effect [63]. Rather, the characteristics of counties with a high asthma burden can be determined.

### 2.2. Study Location

**Figure 1:**
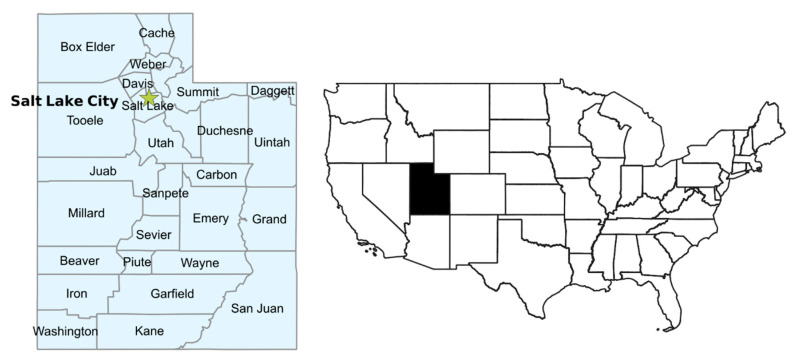
Map of the state of Utah showing county boundaries (**left**) and context within wider USA (**right**).

UT is located in the American Mountain West and is divided into 29 counties (Figure 1). Two-thirds of the population live in highly-populated narrow urban valleys along the Wasatch Front in Salt Lake, Cache, Davis, Weber and UT Counties in the northern part of the state. Metro- and micro-politan counties predominate the northern half of the state with non-metropolitan or rural counties in the south (see Supplemental Material Section 3.1 for definitions). The total population of UT is approximately three million, with almost one million children [64].

### 2.3. Data Collection and Description

County-level data (*n* = 29) were collected for the study. Two health outcome variables: AER visits and AAP and several risk factor variables: mine area, total number of mines, wind erosion risk, % unemployment, % poverty, MHHI, % Native American population, % obesity, HII, annual daily PM_2.5_ concentration, number of red air days per year, AI, elevation, population density, adult smoking rates and % uninsured (Table 1) were included in the study. The health outcome data were age-adjusted (Table 1) to account for differences in the proportions of different ages in the population at risk of asthma.

Data for AAP, smoking and obesity came from the Behavioral Risk Factor Surveillance System (BRFSS) pooled county-level survey data. The BRFSS comprises an annual random digit-dialing telephone survey of American adults administered by the CDC that focuses on health behaviors, lifestyles and diseases [7]. The 2012 BRFSS AAP estimates included 180,500 individuals with current asthma [1] sampled from over two million UT adults. Data for AAP, smoking and obesity were self-reported from a sample of residents from each county [65,66]. Details of the survey questions are given in the Supplemental Material.

ER visits with asthma as the primary diagnosis for the visit and regardless of admission status were obtained from an electronic centralized database for all UT hospitals and arranged into county-level data by patient residence [67]. The percentage of people without health insurance and the average annual PM_2.5_ daily density were obtained from the County Health Rankings website [25]. County-level average annual daily density of PM_2.5_ (CL PM_2.5_, *n* = 29) was generated by the Environmental Public Health Tracking Network (EPHT) through a combined sensor measurement and modeling approach [25,68] and made available via the UT health rankings website [25]. The data come with a detailed description of data limitations which is given in the Supplemental Material. In addition to the CL PM_2.5_ data, daily PM_2.5_ data from the EPA’s downscaler model [69,70,71,72] for each census tract in UT (CT PM_2.5_) (*n* = 588) for 2011–2014 were obtained from the website given in Table 1. Mean, maximum and standard deviations of these data for each census tract were calculated for the summer (May–September) and winter (October–April) months.

Health insurance data included Medicaid (Federal program for those with low income), coverage through commercial and private insurances, and insurance through employment. However, since the Medicare Federal program provides universal coverage for seniors over 65 years, these data were excluded so access to healthcare services could be studied relative to the asthma burden. Data were obtained by survey through the Small Area Health Insurance Estimates (SAHIE) and modeled to provide more stable rates in smaller areas [25]. Data on poverty, unemployment, MHHI and the Native American population were obtained from the Health Indicators Warehouse [66], but the latter was generated by the US Census Bureau (Table 1). The UT Department of Health HII [55], is based on 9 socio-economic indicators detailed in Supplemental Material Section 2.3. Per county median elevations (m above sea level) were derived from a 30 m digital elevation model (DEM) made by the United States Geological Survey (USGS) shuttle radar topography mission (SRTM v3) [73].

The aridity index (AI) was calculated using the following equation:

(1) $$AI=\frac{MAP}{MAE}$$

where MAP is the mean annual precipitation and MAE is the mean annual potential evapotranspiration. County-level maps were generated for median aridity using data obtained from the Global Aridity Potential Evapotranspiration (PET) database using data averaged over the period 1950–2000 [74]. The AI calculation methodology used here produces higher AI values for higher humidity and lower values for higher aridity values [74].

Of the health outcome variables, AER visit rates came in smoothed form, whereas AAP rates were unsmoothed. Smoothing filters unreliable rates for sparsely populated counties from the data by taking rates in adjacent counties into account. For AAP rates, data from three of the most sparsely populated counties—Piute, Daggett and Rich—was missing. Furthermore, due to low numbers of questionnaire responses in primarily rural counties (Beaver, Garfield, Kane, Morgan, San Juan and Wayne), estimates with greater than 30% standard error were deemed unreliable. Therefore, besides combining survey data for the three-year period from 2012 to 2014 to produce more responses overall, AAP rates for these counties were derived from pooled health district figures for the same time-period. UT’s 12 health districts are comprised of groups of two or three adjacent counties.

Total number of mines per county was based on information provided by Bon and Krahulec [16]. Estimated mine area and wind erosion risk were based on ranks of area with mines and a high wind erosion risk in each county based on maps in publications by Krahulec [75] and Dunway et al. [47], respectively. Average windspeed data were obtained from the website given in Table 1 for cities in UT (*n* = 361) and were ordinary kriged to a 5 km grid. The windspeed data were not used in comparison tests and regression analysis.

**Table 1:** Variable descriptions, dates and sources. All data is county-level unless otherwise stated.

Variable	Description	Years	Source
**Health outcome variables**			
Adult asthma prevalence * (%) (AAP)	Current doctor-diagnosed adult asthma	2012–2015	BRFSS, UT Department of Health [65]
Asthma emergency room visits * (AER)	Rate of emergency department visits for asthma per 10,000 population	2012 **	Centers for Disease Control and Prevention [67]
**Environmental variables**			
Estimated mine area	Ranks of mining area by county based on UT Mining Districts Map Image, from UGS publication OFR-695	2018	UT geological survey Krahulec [74]
Median/Minimum aridity index (AI)	County-level AI derived from median pixel from high-resolution global AI map	1950–2000	Global Aridity and PET Database [73]
Median elevation (m)	Median elevation by county calculated from 30 m SRTM data	2000	USGS [72]
Particulate matter (PM_2.5_) (µg m^3 −1^)	County-level: Average daily density of fine particulate matter (PM_2.5_) Census tract: modelled Daily PM_2.5_ levels	2011 ** 2011–2014	County Health Rankings [25] https://healthdata.gov/dataset/daily-census-tract-level-pm25-concentrations-2011-2014.
Red air days per year	red air = daily average PM_2.5_ >35 µg m^3 −1^ (NAAQS standard)	2011	Health Indicators Warehouse (HIW) [66]
Total mines	All types of active mine	2009	Bon and Krahulec (2009) [16]
Wind erosion risk	Ranking of area of county with high erosion risk based on Figure 1e	2019	Dunway et al. (2019) [47]
Wind speed	Average wind speed (mph)	2010–2014	http://www.usa.com/rank/UT-state--average-wind-speed--city-rank.htm
**Health behavior factors**			
Obesity (%) Smoking *(%)	Based on BRFSS height and weight questions (BMI) Current smoking — BRFSS	2009 2011	Health Indicators Warehouse (HIW) [66] County Health Rankings [25]
**Socio-Economic factors**			
Health Improvement Index (HII)	Composite socio-economic index based on 9 indicators for USAD	2018	https://ruralhealth.health.UT.gov/portal/health-improvement-index/, UT Department of Health [55]
Median Household Income ($) (MHHI)	Median household income in dollars	2010	Health Indicators Warehouse (HIW) [66]
Native American Population	Percentage Native American population	2000	US Census data, HIW [66]
Population density	Number of people per square mile, <7 considered highly rural [49]	2010/2011	US Census data, HIW [66], County Health Rankings [25]
Poverty (%)	Estimated by Census Bureau based on data for the SAIPE program	2007/2011	Health Indicators Warehouse (HIW) [66]
Uninsured (%)	Percentage of the population under age 65 with no health insurance coverage	2011	County Health Rankings [25]
Unemployment 16+ (%)	LAUS data come from the CPS, the official measure of the labor force for the nation	2008	Health Indicators Warehouse (HIW) [66]

* rates are age-adjusted, ** data smoothed, USAD Income and Poverty Estimates (SAIPE) program, Local Area Unemployment Statistics (LAUS), Current Population Survey (CPS).

In general, most of the factors used in this analysis will not vary temporally in a way that will significantly impact the outcome of this study. However, there are some factors, such as construction in rural areas, which will slowly start to affect the outcomes of the study.

### 2.4. Statistical Analysis

Comparison tests (Mann–Whitney U and Kruskal–Wallis H) were conducted using the AAP and AER visit rates as grouping variables. For Mann–Whitney U tests, two groups of AAP and AER visits, low (L) and high (H), were used with the threshold between groups of 10% and 25 per 10,000, respectively. For Kruskal–Wallis H tests, three groups of AAP and AER visits were used, L, medium (M) and H with the thresholds between groups of 8 and 10% for AAP and of 20 and 30 per 10,000 for AER. The characteristics of the counties with H, L and H, M, L rates of AAP and AER visits were determined by investigating the mean ranks of each risk factor variable for each type of county). The Statistical Package for Social Sciences (SPSS, version 24) was used for comparison tests. Non-parametric comparison tests were chosen so that any non-normality of the variables and unequal-sized groupings following natural breaks would not be an issue.

Pearson correlation analysis was used to select variables for regression analysis, by identifying the variables with the strongest correlations with AAP and AER visits, redundant variables and to identify multiple collinearity between independent variables. Associations between AAP and AER visits and the risk factor variables were investigated using multiple linear regression (MLR) (Tables 3 and 5). Regression analyses were used to investigate the most significant factors associated with AAP and AER visits in UT. A best sub-set approach was used for MLR using SpaceStat [76].

Regression kriging involves using the degree of spatial autocorrelation and the relationship between sparse and dense data to improve the density of information for the sparse variable by predicting it at locations where it is unknown. A regression model was fitted between sparse (county-level data, e.g., AAP and AER rates, *n* = 29) and more dense data (USAD e.g., HII *n* = 99 or census tract data, CT PM_2.5_ *n* = 588) aggregated to the county-level. A variogram was then computed from the residuals of this regression and the residuals were ordinary kriged to the USAD or census tract locations. The equation of the regression model (see the Supplemental Material for equations and more details) was then used to regress values at the USAD and census tract locations, and these regressed values and the ordinary kriged residuals were summed to give the regression kriged (RK) estimates at the USAD locations. For more information on regression kriging, see Hengl et al. 2007 [77]. Root mean squared errors (RMSE) for the aggregated regression kriged data were calculated based on comparison with the original county level data.

The local Moran’s I (LMI) [78] is a statistic that compares each county-level variable with the average rate recorded in neighboring counties (*n* = 5 here) to test for the presence of significant positive (spatial clusters) or negative (spatial outliers) spatial autocorrelation. Determining whether the local autocorrelation is significantly different from zero requires knowledge of the distribution of LMI values under the null hypothesis of spatial randomness. Monte Carlo simulation is used to repeatedly (999 times) randomly shuffle all the AAP or AER data and the distribution of simulated LMI values is then compared with the sample value computed from the data to calculate the *p*-value of the test. Where *p* < 0.05 indicates that the corresponding county is either: (i) a significant spatial outlier (HL: high value surrounded by low values (pink) and LH: low value surrounded by high values (pale blue) or (ii) part of a cluster (HH: high value surrounded by high values (red), and LL: low value surrounded by low values (blue). The Simes correction was used to correct *p*-values for multiple testing because significance testing of the LMI for each county increases the number of tests and therefore the risk of false positives [79]. The LMI was used to identify significant clusters of particularly high or low AAP rates (univariate LMI) and AER visits. LMI analysis was completed in SpaceStat [76].

Moving correlation analysis was performed on the RK USAD and CT AAP and AER data with some predictor variables that were also available at these higher densities. Moving correlations use a moving window approach to correlation and were performed using the *n* = 20 nearest neighbors with equal weight given to each neighbor in SpaceStat [76].

## 3. Results

### 3.1. Descriptive Statistics and Spatial Patterns of Variables

Means, medians and histograms were computed for each variable (not shown) and these showed that the health outcome variables (AAP and AER rates) had a relatively normal distribution with skews close to zero. Skews for the risk-factor variables were generally within the range −1 to 1 so they were not transformed for Pearson correlation and regression analyses. However, population density and red air days were transformed to logarithms because they had skews of 3.351 and 1.29, respectively.

**Figure 2:**
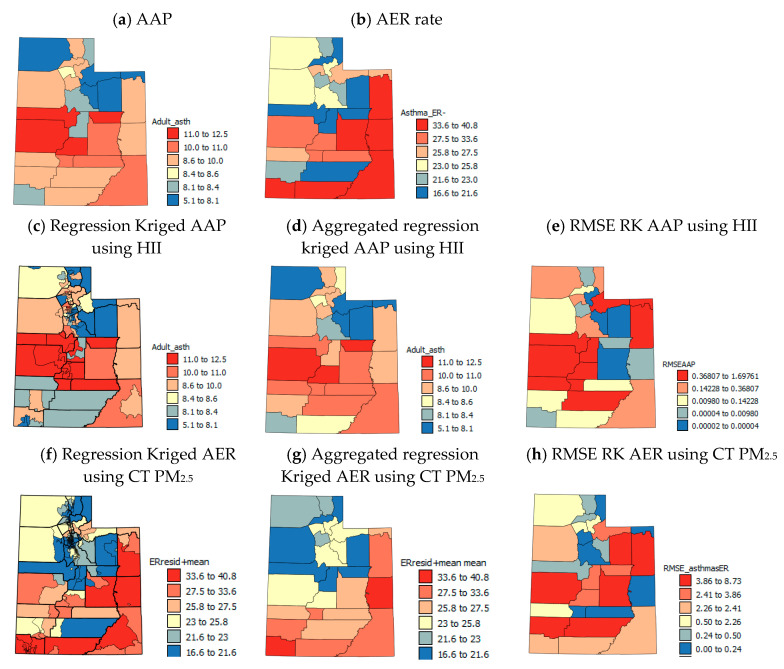
Maps of health outcome variables.

**Figure 3:**
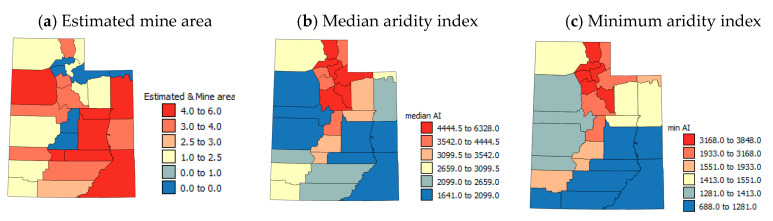

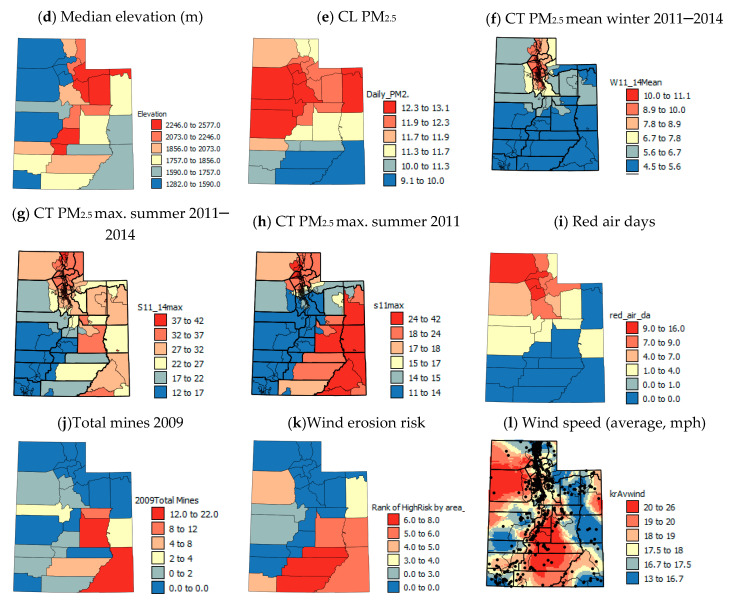
Maps of environmental variables.

**Figure 4:**
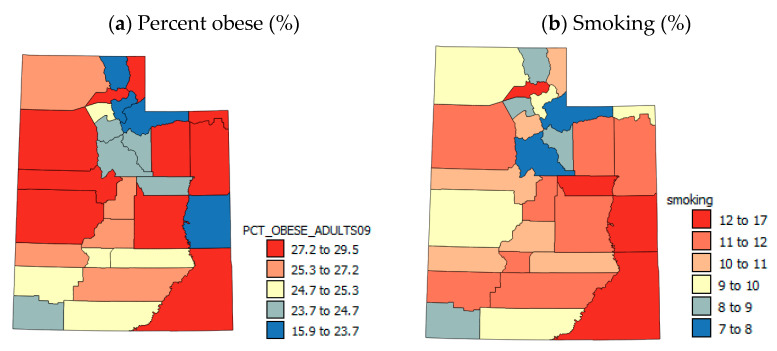
Maps of health behavior variables.

**Figure 5:**
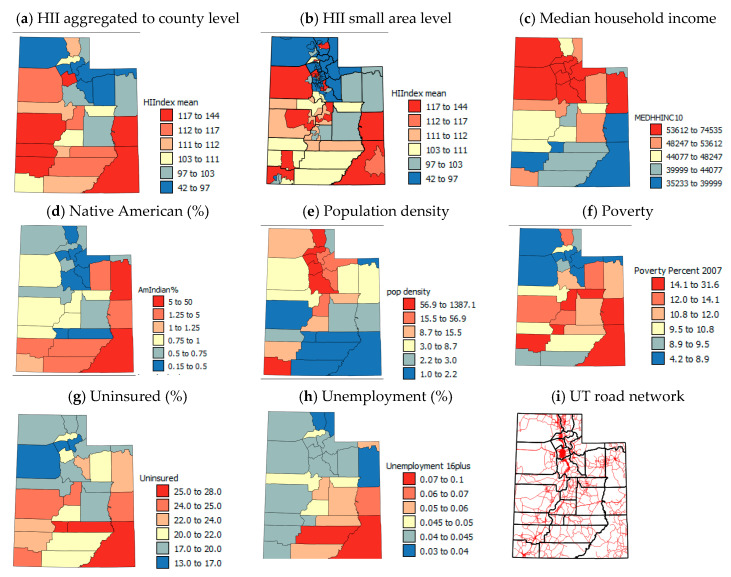
Maps of socio-economic variables.

Figure 2, Figure 3, Figure 4 and Figure 5 show the spatial patterns in the health outcome, environmental, health behavior and socio-economic variables at the county-level within the state of UT. AAP rates (Figure 2a) are lowest in northern UT with the highest rates in the central and southern UT and medium rates in the populous northern counties (Cache, Morgan, Salt Lake and Weber, UT, USA). Patterns in AER visits (Figure 2b) show almost alternating west–east stripes across the state of high and low rates in southern and central UT and generally more medium rates in northern UT.

The estimated mine area is largest in south and eastern counties of UT and in Tooele county (Figure 3a) whereas the total number of mines is highest in south eastern UT (Figure 3j). Low median AI values suggest that a county is generally dry, but low values of minimum AI shows that a county has some very dry locations. Generally, the central western counties and southeastern counties (Figure 3b,c) are driest and northern counties are moister. The moister counties also tend to have higher elevations and there are north to south and east to west decreases in elevation (Figure 3d).

The maps of CL PM_2.5_ and red air days (Figure 3e,i) are similar with generally higher values in the north of the state, medium values in central areas and low values in the south. The main difference is high levels of PM_2.5_ in Tooele, Juab and Millard counties. These are no heavily populated counties like others with high PM_2.5_ levels and numbers of red air days, suggesting that dust could come from dried lake beds in these counties and the Great Basin Desert in Nevada to the west [28], as well as the Bingham open pit copper mine which is the deepest open pit mine in the world and is close to Tooele.

**Figure 6:**
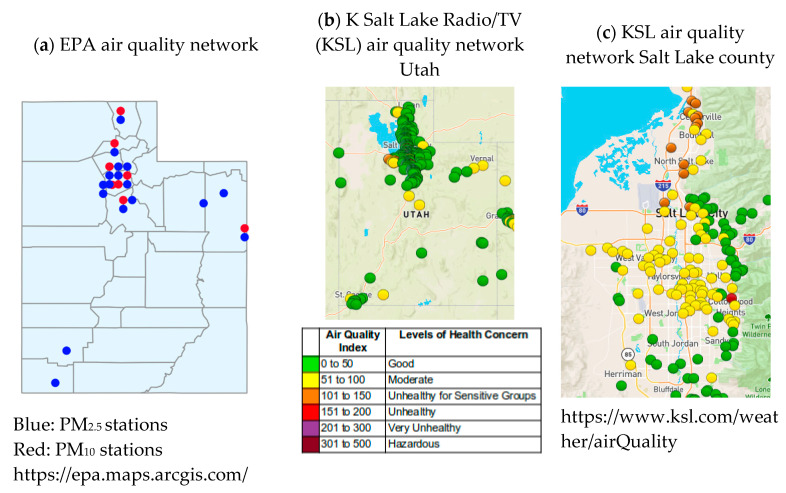
Maps showing locations of (**a**) EPA and (**b**) KSL Air quality monitoring stations within Utah and (**c**) Salt Lake County.

Figure 3k shows that the wind erosion risk is high for Tooele county but is low for Juab and Millard, suggesting that a large portion of the PM_2.5_ burden in these counties comes from Nevada, or Tooele county. The average wind speed for Tooele county is also high, but it is lower for Juab and Millard counties (Figure 3l). The wind erosion risk (Figure 3k) of sediments is high in the southeastern counties of UT but CL PM_2.5_ values (Figure 3e) are not high, and this may be due to higher average wind speeds (Figure 3l) in central, rather than southern eastern UT. It may also be due to the lack of PM_2.5_ monitors in southern UT (Figure 6a), causing greater uncertainty in actual predicted levels as well as wind-eroded sediments tending to be PM_10_ size. Figure 3f–h show the CT PM_2.5_ values separated by season. The mean winter values for 2011–2014 (Figure 3f) show a pattern of the highest levels of PM_2.5_ being in the most populous census tracts and counties in central parts of northern UT. This suggests that on average annually, the EPA models that are based on simulations from monitor and weather data are predominantly showing patterns of population density (Figure 5e) and traffic (Figure 5i). This would, however, be expected, as the monitoring stations are concentrated in urban areas (Figure 6a). The summer maximum PM_2.5_ maps (Figure 3g,h) show high PM_2.5_ levels in the most populous counties in northern UT and in the counties with most mines/mine area (Figure 3a,j) and wind erosion risk (Figure 3k) in south eastern UT. This suggests that the high max. CT PM_2.5_ levels in south eastern UT in the summer could be a result of wind erosion and mine dust.

Obesity and smoking (Figure 4a,b) show some similar patterns with generally low rates in the northern UT and higher rates in the south and east. There are some local differences, suggesting that there may be large differences within the most populous northern counties associated with socio-economic variables.

The HII (Figure 5a) is calculated using MHHI, poverty and unemployment rates, so similarities for these variables (Figure 5c,f,h) are expected. The northern and north eastern counties tend to have the lowest levels of deprivation and the south higher levels. However, in the central western counties, while poverty levels are high and MHHI are low, the unemployment rates are low, suggesting that most people are employed in low-paid jobs. The USAD for the HII (Figure 5b) show that there are large within-county differences in HII, so when averaged to the county-level, information about within-county variation is lost. The other socio-economic variables, Native American population, population density and % uninsured (Figure 5d,e,g) show broadly similar patterns to the variables included in the HII (Figure 5a,c,f,h), with low proportions of Native American population, high population densities and low %uninsured in the north and higher proportion of native Americans, uninsured and lower population densities in the south. An exception to these trends is Washington county and more particularly the St. George area, a retirement area in the southwestern corner of the state which tends to be less economically challenged than neighboring counties. San Juan and Juab counties seem to be the worst off economically in the state.

### 3.2. Adult Asthma Prevalence: Comparison Tests, Correlation, Regression and Spatial Analysis

The results of Mann–Whitney U for AAP are shown in Table 2. There was a significant difference (*p* < 0.05) between counties with low and high rates of AAP in terms of several risk factor variables: estimated mine area, median and minimum AI, red air days, total mines, wind erosion risk, median household income, population density and poverty. The most significant differences between high and low AAP counties were for Median AI and poverty. The Mann–Whitney U tests suggest that counties with high AAP rates are characterized by a large estimated mine area and total number of mines, low AI meaning they are arid, have a high wind erosion risk and have a low number of red air days. These counties also have low MHHI and population densities and higher levels of poverty. Other variables that were close to significant *p* = ~0.1 suggest that these counties also have higher obesity, smoking, unemployment and uninsured rates.

For Kruskal–Wallis H tests, AAP was divided into counties with low, medium and high rates (Table 2). The variables that showed significant differences (*p* = 0.05) between these groups of counties were estimated mine area, median AI, total mines, wind erosion risk, poverty and the HII. Some variables that showed significant (*p* = 0.05) differences between groups of counties in Mann–Whitney U tests were among the variables that were close to significant *p* = ~0.1 in the Kruskal–Wallis H tests such as minimum AI, median elevation, smoking, MHHI, and population density. The Kruskal–Wallis H tests suggested that the counties with high rates of AAP are characterized by a large mine area and number of mines, are arid and have a high wind erosion risk, are at medium elevations and have a low number of red air days. They also have high smoking and poverty rates, HII, low MHHI and low–moderate population densities.

The variables that show significant *p* < 0.1 Pearson correlations with AAP (Table 2) are mostly the same as the variables that showed significant differences between AAP groups in the comparison tests namely: mine area and number of mines, median and minimum AI, wind erosion risk, obesity, smoking, poverty and HII. The strongest and most significant correlations (*p* = 0.01) were with estimated mine area, median AI, wind erosion risk, obesity and HII. All variables showed the expected sign of correlation and patterns in mean ranks (Kruskal–Wallis test) with AAP given the conceptual model apart from population density, CL PM_2.5_ and red air days, which all showed unexpected negative correlations with AAP.

The comparison and correlation tests for AAP (Table 2) suggest that longer-term environmental (e.g., mine area, median aridity and wind erosion risk) and socio-economic characteristics (HII, poverty) are most important to AAP. Table 3 shows the results of best-subset regression for AAP. The correlations of risk factor variables amongst themselves are shown in Table S1 of the Supplemental Material. The socio-economic variables were moderately (r = ~0.3–0.5) correlated amongst themselves so they were included in multiple runs of the sub-set approach one at a time to avoid multi-collinearity issues. By far the strongest correlation between risk factor variables was for population density and red air days (*r* = 0.748), so both of these variables should not be included in the same regression model if multi-collinearity is to be avoided. The best-subset regression model for AAP had an R^2^ = 0.425 and included median AI, total mines, smoking and Native American population in the model, while most of the variables were close to significant at *p* = 0.05, only native American population was significant at *p* = 0.05. The regression model showed that areas with high AAP are generally arid, have lots of mines, high smoking rates yet lower native American populations. These characteristics seem to be somewhat synonymous with central counties of the state along and west–east axis from Juab and Millard across to Grand county (Figure 2a).

### 3.3. Spatial Analysis to Investigate the Reasons for Some Unexpected Correlations with AAP at the County Level

The unexpected correlations with AAP for population density, CL PM_2.5_ and red air days are thought to be a function of several factors which will be investigated via spatial analysis of sub-county level data. First, the global correlation coefficient *r* does not consider spatial variation in the relationship between two variables. Second, pollution is quite a localized phenomenon spatially, as is AAP, so CL AAP, PM_2.5_ and red air days erase local, within-county differences that could show the expected positive correlations between AAP and air pollution. Next, UT can be divided into two (north and south) in terms of many of the environmental and socio-economic variables investigated. In the more heavily populated north, traffic pollution dominates (PM_2.5_), whereas in the south, dust from arid areas and mines dominates pollution (PM_10_). There is also an issue of seasonality in the patterns; the PM_2.5_ from traffic pollution is a constant problem that is exacerbated in winter temperature inversions, whereas dust storms largely occur in the summer months when it is dry. An additional issues is that the PM_2.5_ data for the south are based on a very limited number of monitoring stations (Figure 6a,b) and most of the values are modelled based on measurements from stations very large distances away and PM_10_ levels are not monitored at all by the EPA in southern UT (Figure 6a). Finally, socio-economically, southern counties are generally more depressed compared to the north and asthma prevalence is greatest in the lowest socio-economic group for every state in the USA [56]. Poorer living conditions, diet, healthcare and manual labor in dusty environments such as mines can all contribute to increased AAP in lower socio-economic groups. At the county-level, the socio-economic differences that exist in the north of the state between USAD units within the same county are lost.

To illustrate that sub-county (USAD level, *n* = 99) AAP, if available, would show large within-county variations in northern UT like the USAD HII data [55] that are smoothed out by averaging (Figure 5a,b), the USAD HII data were used for RK of AAP data to the USAD locations. Details of the RK process used are given in Supplemental Material Section 3.1. The RK AAP values were aggregated to the county-level (Figure 2d) and correlated with the original CL AAP values (Figure 2a). The correlation coefficient was *r* = 0.93 and the mean RMSE (Figure 2e) was 0.35%, showing the good accuracy of RK. Figure 2c,e show, as suspected, that within-county boundaries there are USAD units with very different AAP, but aggregating these data to the county-level (Figure 2d,g) smooths out values by averaging. As county-level data on air pollution (PM_2.5_ and red air days) are annual averages and are based on sensor data and modeling, there is more uncertainty in the PM_2.5_ data for southern UT due to lack of sensors (Figure 6a). In northern UT, just as with the HII data, there can be large differences in air quality within-counties which can be seen by the different colors of the symbols for monitoring stations that are close to one another in Figure 6c. These spatial differences are masked at the county scale and average PM_2.5_ levels are reported for counties where there are large within-county differences. Temporal patterns in CT PM_2.5_ levels show seasonality in the spatial patterns (Figure 3f–h) with constant high levels of PM_2.5_ in the north, but high levels of PM_2.5_ in the east and south of the state.

**Figure 7:**
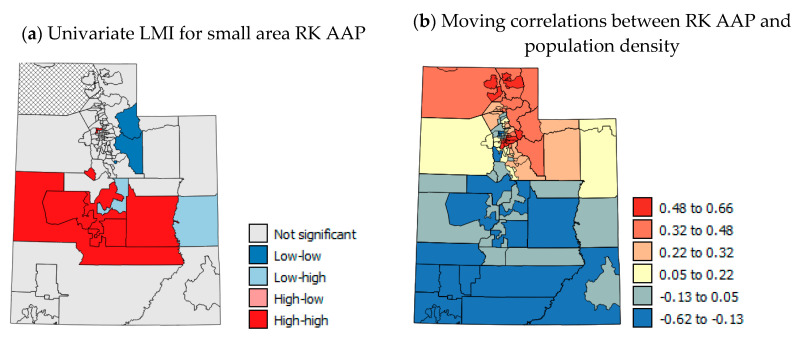

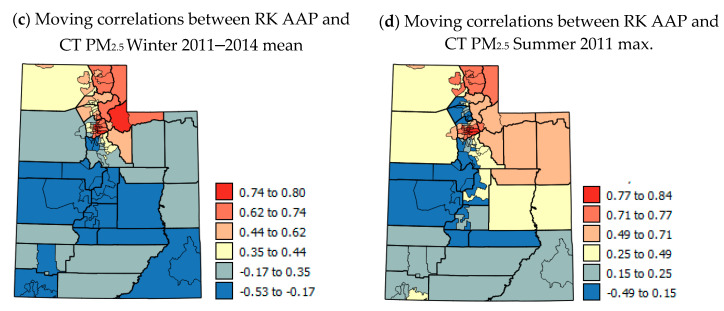
Local Moran’s I (LMI) and moving correlation analysis for RK AAP and other variables.

Figure 7a shows the univariate LMI results for RK AAP with a statistically significant cluster of high rates across central UT. Figure 7b–d show moving correlation maps for the USAD level. These show strong localized positive and negative correlations. The correlations between AAP and population density, and CT PM_2.5_ for summer 2011 max and winter 2011–2014 mean show distinct patterns of positive correlation in northern UT and negative correlations in southern UT (Figure 7c–e). This shows that in northern UT there is an expected positive relationship with population density and PM_2.5_ when USAD are investigated. The positive local correlations with PM_2.5_ suggest the effect of traffic-generated pollution on increasing AAP in the north, whereas the negative correlations in southern UT suggest that other factors are more important such as socio-economic and lifestyle factors as well as wind erosion risk and mines. The findings of moving correlation analysis were further confirmed by correlation analysis of the USAD for metro and non-metro areas (Table 4). Northern UT includes most of the Metro areas in the state and southern UT most of the non-Metro areas (see Supplemental Material Section 3.1 for definition and designations). Table 4 shows correlations between RK AAP and CT PM_2.5_ that were aggregated to the USAD level. The correlations with winter 2011–2014 mean, summer 2011 max and summer 2014 max were all weakly positive when considering all USAD areas (*r* = 0.022–0.273, Table 4), however, the correlations for metro areas were moderately positive (*r* = 0.378–0.428, Table 4) and those for non-metro areas were negative or weakly positive (*r* = −0.161–0.211). This suggests that factors other than patterns of PM_2.5_ pollution are important to AAP in non-metro counties and that there is a north–south divide in causes of AAP in northern and southern UT.

### 3.4. Asthma ER Visits: Comparison Tests, Correlation and Regression Analysis

The results of Mann–Whitney U tests for AER visits are shown in Table 5. There was no significant difference (*p* < 0.05) between counties with low and high rates of AER in terms of any of the risk factor variables, but red air days was significant at *p* = 0.1 and median AI, population density and uninsured were the variables had *p*-values between 0.1 and 0.2. Although there is generally lower significance than for AAP, the Mann–Whitney U tests suggest that counties with high AER rates tend to be arid, rural areas with high % uninsured individuals and few red air days.

For Kruskal–Wallis H tests, AER visit data were divided into counties with low, medium and high rates (Table 5). The variables that showed significant differences (*p* = 0.05) between these groups of counties were minimum AI, red air days, wind erosion risk and smoking. There were also significant differences between county groups at the *p* = 0.1 level in terms of mine area, median AI, PM_2.5_, mines, MHHI and % uninsured. The Kruskal–Wallis H tests suggest that the counties with high rates of AER visits are characterized by a large mine area and number of mines, are arid and have a high wind erosion risk, and have low PM_2.5_ levels and low numbers of red air days. They also have high smoking and uninsured rates, and low MHHI. For AAP, the mean ranks of risk factor variables showed an order with the rates of AAP for the three groups, suggesting either a positive or negative relationship between the variables. However, for AER visits, the group of counties with medium rates had the lowest mine area/number of mines, were moister (less arid) and had the least wind erosion risk and experienced the highest PM_2.5_ levels and numbers of red air days. These counties also had the lowest rates of obesity, smoking, poverty, uninsured and unemployment and had the highest MHHI and population densities. These patterns suggest that aridity, dust and occupational and general exposure to mine dust as well as greater socio-economic problems in rural areas result in the highest AAP and ER rates being in such counties, but the wealthier urban counties in the north of UT that experience most vehicular air pollution and hence high PM_2.5_ levels and numbers of red air days have medium AER visit rates. This reveals an issue that is masked in Mann–Whitney U tests when counties are only split into two groups based on AER visits. As county-level data are more readily available than USAD, in densely populated counties there can be neighboring smaller geographic areas with very different socio-economic and pollution characteristics. The HII was available for USAD locations and Figure 5b shows that in densely populated northern UT areas with large differences in socio-economic characteristics are found within the same counties. It is likely that these areas also have large differences in pollution and AAP/AER rates.

The variables that show the most significant *p* < 0.1 correlations with AER rates (Table 5) are mostly but not exclusively the same as the variables that had the lowest significance values for the Kruskal–Wallis H tests, namely: median and minimum AI, wind erosion risk, PM_2.5_, native American population, and % uninsured. The strongest correlation was with wind erosion risk. As for AAP, the correlations of PM_2.5_ and red air days with AER visits were negative, which is unexpected given the conceptual model, but the order of mean ranks for the H, M and L risk groups suggests some AER rates for heavily populated and polluted counties come out as medium rates when there are probably more local patterns of high rates associated with high pollution levels and low socio-economic status.

The comparison and correlation tests for AER visits (Table 5) suggest that environmental variables that can show periodic temporal changes (e.g., minimum aridity, PM_2.5_ and wind erosion risk) and lack of health insurance are most important to AER visits. Given the differences noted between metro and non-metro (north and south) counties for AAP, correlations for metro and non-metro areas were investigated and can be found in Tables S2 and S3 of the Supplemental Material. Most notable of these was that the correlation between AER visits and median AI was −0.39 (*n* = 29) for all counties (Table S1 Supplemental Material) but was −0.715 for non-metro counties. This suggests that desert dust may be more of an issue in AER visits in the non-metro counties that are predominantly in the south.

Table 6 shows the results of best subset regression for AER visits. The same precautions were taken in this regression as for that for AAP to avoid issues of multicollinearity. The R^2^ value was low at 0.307 and only wind erosion and % uninsured were included in the model, but only wind erosion risk was significant.

### 3.5. Spatial Analysis of AER Data

The unexpected correlations of PM_2.5_ and red air days with AER are likely to stem from similar sources to the unexpected correlations of these variables with AAP. The AER data were RK with CT PM_2.5_ data (summer 2011 max. *r* = 0.323 and winter 2011–2014 mean *r* = −0.347, see Supplemental Material Section 3.1 for full details of the RK process). When the RK AER data (Figure 2f) were aggregated to the county-level (Figure 2g), the correlation with the original county-level AER data was *r* = 0.876 and the mean RMSE (Figure 2h) was 2.197.

**Figure 8:**
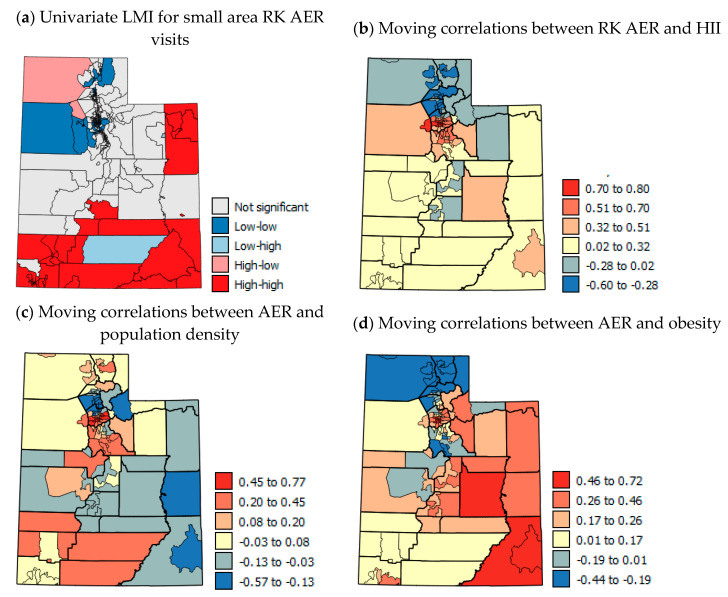
Local Moran’s I (LMI) and moving correlation analysis of RK AER visits and other variables.

Figure 8a shows the univariate LMI results for RK AER with a statistically significant cluster of high rates across southern UT and another cluster of high rates in the eastern corner of the state. Figure 8b–d show moving correlation maps for the USAD level. These show strong localized positive and negative correlations. Not all variables were available at the USAD level and so moving correlations could not be calculated for each variable. The patterns in moving correlations with AER visits are more complex than for AAP (compare Figure 7b–d and Figure 8b–d). The correlations with HII (Figure 8b) are negative in the far north of the state, then are positive in the most populous areas near Salt Lake City and in south eastern UT. This suggests that socio-economic considerations are most important to AER visits in some of the most populous areas and south eastern UT. The correlations of AER with population density (Figure 8c) show several counties in the north and south that have positive correlations. This suggests that higher AER rates are linked to urban areas in these counties and with rural areas where negative correlations occur (mainly in central, south and eastern UT). Correlations between AER visits and obesity are positive across central, eastern and south eastern UT, whereas negative correlations are concentrated in the far north of the state. These results suggest that the clusters of high AER visits in the south and eastern areas of the state may be linked to rurality and lack of regular doctor visits to keep symptoms under control, which would help in avoiding AER visits. However, the HH clusters of USAD areas also correspond with patterns of summer CT PM_2.5_ levels and high wind erosion risk (compare Figure 8a, Figure 3g,h,k).

### 3.6. Summary of Results for AAP and AER

Table 7 and Table 8 show summaries of all of the results for AAP and AER. The first column shows whether the expected association between AAP and AER and a given variable should be positive (red) or negative (blue) based on the literature review. The other columns show for the various methods used whether the association with AAP and AER was positive (red), negative (blue) or mixed (yellow). An indication of the level of significance of each variable in each test is also given. Overall, the results for AAP showed the expected associations in comparison and correlation tests apart from for elevation, population density, red air days and PM_2.5_. For elevation, this was probably because none of the tests showed significant results as most of the state is considered high elevation, and once above a certain threshold, further increasing elevation does not have marked effects. For population density and PM_2.5_, small area and temporal data were able to show that associations in metro and northern areas were positive (as expected) and more significant, particularly in winter. In contrast, patterns for southern and non-metro areas tended to be negative. Overall, the most important factors to AAP seem to be wind erosion risk, mining activities, aridity and long-term socio-economic characteristics, as shown by the HII.

Table 8 shows that the association between risk factors and AER is more complex. Several of the variables showed unexpected associations in Mann–Whitney U tests. Many of the variables are colored yellow for the Kruskal–Wallis H tests. For variables such as PM_2.5_ and population density, medium levels of PM_2.5_ and population density were associated with high AER. This suggests that moderately sized towns with moderate levels of PM_2.5_ pollution from traffic have the highest rates of AER visits. Moving correlation analysis with sub-county level data showed areas with positive and negative associations with AER in the north and south probably associated with local differences in pollution exposure as well as differences in socio-economic characteristics. In terms of consistency of association and significance, the factors that are important to AER are aridity, wind erosion risk, mining, elevation, native American population, %unemployed and %uninsured with wind erosion risk and uninsured being the most important factors. These are more temporally transient factors which suggest greater AER during dust storms and a higher proportion of uninsured people presenting at the ER with asthma symptoms. These potential effects need confirmation with studies of temporal and individual level AER data.

## 4. Discussion

The comparison tests highlighted the importance of socio-economic factors as well as environmental and health behavior factors to AAP and AER visits (Table 2 and Table 5). Environmental variables such as minimum AI, red air days and wind erosion risk, which could be associated with specific events, showed more significant differences with and stronger correlations with AER groups than AAP groups in Kruskal–Wallis tests and correlation analysis. Economic variables generally showed more significance between groups in comparison tests and higher correlations with AAP than AER visits, apart from % uninsured. This makes sense given that those that are uninsured are more likely to present at the ER in case of an asthma attack as they may not have regular prescriptions for management of asthma symptoms in place. However, the other socio-economic variables are more related to long-term characteristics of socio-economic disadvantage, which is reflected by HII being the most strongly correlated socio-economic variable (Table 2). National figures show for every state that the lowest income group has the highest AAP [56]. This suggests greater residential and occupational exposure to pollutants and irritants in poor quality housing and locations and more manual labor as well as greater obesity and smoking levels etc. may contribute to higher AAP among groups with lower socio-economic status [55,61].

The large separation distances between monitoring stations in southern UT (Figure 6a) leads to large uncertainty in the air pollution data for large regions of southern UT, suggesting that levels of desert and mining dust are not being effectively monitored or characterized. Levels of this type of atmospheric pollution (larger particles, PM_10_ rather than PM_2.5_) are likely to be greatest when winds are high, and in the summer when the soil is driest and most mobile. This was shown by the difference in patterns of the mean winter PM_2.5_ levels and summer max PM_2.5_ levels at the census tract level. The latter showed higher levels in south east UT, which are unlikely to be due to traffic pollution. In contrast, CT PM_2.5_ levels in the north are more constant due to traffic, but are likely to peak in calm, temperature inversion conditions in the winter. Dust storm events tend to have extremely poor air quality, but as the CL PM_2.5_ data in this study are annual averages, the effects of such events are not evident when comparing pollution levels between counties. Figure 6a shows the active PM_2.5_ and PM_10_ monitoring stations in the EPA’s network in UT and it shows that there are very few PM_2.5_ stations in southern UT and no PM_10_ stations. The differences in the type of air pollution in the north and south of the state need to be appropriately addressed by installing PM_2.5_ and, especially PM_10_ monitoring stations in mid-sized towns separated by vast distances in the south of the state; since only sparse data are available for southern UT, it is impossible to assess the accuracy of models that are used to predict pollution levels across these vast areas of the state.

The findings that counties with high AAP rates were significantly more arid (lower median AI) (*p* = 0.01), had significantly higher wind erosion risk (*p* = 0.05), larger estimated mine areas (*p* = 0.05), and more total mines (*p* = 0.05), than those with low AAP burdens, are highly suggestive of dust driving up AAP in high burden areas. Additionally, median AI and total mines featured in the best subset regression model for AAP (Table 3). Previous studies [37,38,39,40] focused more on the effects of aridity and dust storms on asthma exacerbation (AER) and there are few comparable studies for AAP. Comparison tests for AER visits followed the same general trend, with wind erosion being significant (*p* = 0.05) and minimum AI significant (*p* = 0.01) in Kruskal–Wallis comparison tests (Table 5); and wind erosion risk (*p* = 0.05) featuring in the best subset model for AER visits (Table 6). Perhaps the high negative correlation found between median AI and AER visits in the non-metropolitan counties (r = −0.715, *p* < 0.01) (Table S3 Supplemental Material) where PM_2.5_ come from vehicle emissions is largely taken out of the equation, best characterizes the relationship and is highly suggestive of the role of desert dust in rural areas of UT in asthma exacerbation. These results contrasted with a Kuwaiti desert study [39], where pediatric AER visits correlated with increases in relative humidity, and not aridity. However, the present study utilized aggregated yearly AER visit data for all ages and visits were not linked temporally and, therefore, the ecological fallacy cannot be ruled out. Although aridity is a meteorological feature that is generally fixed, knowledge of this relationship can assist in asthma resource planning. Furthermore, since wind tunnel studies in the UT desert [80] showed desert soils to be relatively stable when undisturbed, upstream policies restricting land development for mining and other human activity adjacent to population centers in desert areas may be warranted. More research, such as the studies of Goodman et al. [28] and Dunway et al. [47], is needed particularly in southern UT to assess the effects and impact of wind erosion, and desert and potentially toxic mining dust, respectively.

The present study, particularly the moving correlation analysis based on RK AAP data, highlights the north–south divide in UT. There are large socio-economic differences between the north and the south and there are different air pollution problems occurring in both areas. Traffic pollution and winter inversions and PM_2.5_ dominate in the heavily populated Wasatch Front northern areas and wind-blown desert and mining dust (probably mostly PM_10_) dominate in southern UT in the summer. The uncertainty of pollution monitoring in southern UT makes relationships with AAP and ER visits and pollution difficult to detect particularly at the county-level. For air pollution exposure, while mean levels per county show the broad trends in exposure, air pollution can show great local heterogeneity [81]. The stark within-county differences shown in USAD HII and CT PM_2.5_ as well as the change in sign of correlations between AAP and PM_2.5_ when CT PM_2.5_ data were used instead of CL PM_2.5_ show the need for data for areas smaller than counties for future spatial studies. RK for pollution levels might be better done conducted satellite imagery as the dense regressor. Other suggestions for future work include adding a temporal component to cast more light on the UT PM_2.5_ situation. Such studies could use satellite imagery to investigate the spatial variation in PM_10_ levels on particularly windy days and PM_2.5_ on red air days. Furthermore, if temporal AER visit data could be obtained, it would be useful to investigate AER visits on days with temperature inversions in northern Utah and on days with high winds in southern Utah.

Johnson and Graham [22] stressed the importance of developing practical evaluative tools that focus on susceptible population groups. Indeed, the effects of PM pollution would have a high impact in the state of UT given large family-size and the high number of children per capita attributable to Latter-day Saint (Mormon) religious influences [82].

The present study did not demonstrate significant differences in elevation between high and low burden counties for both AAP and AER visits, nor did elevation feature in the regression models for either health outcome. However, a significant negative correlation (*r* = −0.31) was found between AAP and elevation supporting evidence from the conceptual model [42,43] that higher altitudes with less exposure to winter temperature inversions may be beneficial for asthma control and development. Studies of adolescent and adult asthmatics [83] also provide evidence of the benefits of short stays at high altitudes above 1500 m to stave off asthma exacerbation, and this may have relevance in UT. A western-US study [84] demonstrated a negative relationship between lung cancer incidence and elevation attributed to a carcinogenic effect of increased oxygen at lower altitudes. Additional research is needed to determine whether a similar aero-toxic effect could explain the negative correlation between AER and elevation observed in the present study. Although elevation is fixed, knowledge that lower elevations are associated with higher rates of AER visits may aid in planning and prioritizing asthma resources and preventive healthcare within UT. The fact that AAP and AER rates were generally higher and showed statistically significant clusters of high rates in the less PM_2.5_ polluted south of the state and the analysis suggested that socio-economic factors and related health behaviors (smoking and obesity) may be more important to asthma studies than previously thought. This finding is backed up by asthma prevalence being greatest in the lowest socio-economic group for every state in the USA [56].

Contrary to the results of cross-sectional [48] and longitudinal studies [49], the results of comparison tests in the present study found no significant differences in smoking between counties with high and low AAP rates, although AAP and % smoking were significantly correlated (*r* = 0.42; *p* = 0.05) (Table 2) and smoking featured in the best subset regression model for AAP (Table 3). Smoking appeared to be less important to AER visits, although Kruskal–Wallis comparison tests showed significantly higher smoking rates in counties with high AER visit rates compared to counties with medium visit rates (Table 4). Smoking rates are generally low in UT and aggregated county-level data may mask patterns at the individual level; furthermore, the present ecological design cannot control for individual level confounders like the “healthy smoker effect” [49]. Despite UT’s low smoking rates, likely attributable to LDS religious influences [82], stop-smoking programs and addiction support groups may be valuable resources to invest in to help lower AAP rates. However, smoking rates are generally higher in rural areas and may reflect a more complex relationship with employment and other socio-economic factors. Correlation analyses revealed a significant positive correlation (*r* = 0.439, *p* < 0.05) between the percentage uninsured and the percentage of adult smokers. In short, commercial health insurance coverage is largely tied to employment in the United States; unemployment affects the social gradient inducing stress, leading to lack of social support and addiction, thereby driving up smoking rates [85]. Therefore, any attempt to reduce smoking and AAP rates needs to be multifaceted and address social determinants of health (SDH).

The finding of higher percentages of uninsured individuals in counties with high AER visit rates, a significant positive correlation between AER rates and % uninsured (*r* = 0.34; *p* = 0.05) (Table 4) and uninsured featuring in the regression model for AER visits (Table 5) is consistent with the findings of previous studies [57,58]. Nevertheless, addressing access to healthcare as a barrier to routine treatments and maintenance medications for asthma may stave off costly emergency room care. Due to the remote nature of desert areas and lack of healthcare facilities, this may be pronounced in the rural areas of UT. Wilkinson and Marmot [85] (p.1) describe ‘universal access to medical care…[as] one of the social determinants of health.’ In addition to access to quality health services, education regarding routine asthma management is an important element of any effort to reduce costly emergency care for asthma exacerbation [1]. The higher 2015 AER visit rate per 10,000 population in males compared to females [1,2] suggests that targeted education sessions could significantly reduce the AER visit burden in this demographic and lead to attainment of AER HP2020 targets.

Although statistical comparison tests suggested that population density was not important to AER visits and AAP, the difference in strength and sign of correlations between metro and non-metro areas is strongly suggestive of the role of emissions, human activity and PM_2.5_ air pollution adding to the UT AAP burden. AAP rates are more likely to be affected by the long-term atmospheric conditions than AER visits which will be more correlated with temporal spikes in atmospheric pollution, such as from winter temperature inversions and wind-blown summer dust. The distance to major roads could also be considered in future studies as well as consideration of other forms of atmospheric pollution.

UT, with only 4 rural counties meeting, and 25 counties exceeding WHO air quality standards, (PM_2.5_ average daily annual concentrations <10 µg m^3^) appears to be faced with an unrealistic upstream goal given the high background levels of desert dust driving up PM_2.5_ levels in rural areas (Figure 3e–h). However, of the 11 counties exceeding NAAQS PM_2.5_ standards (>12 µg m^3^), 7 are metropolitan counties with 1 or more red air day per year, showing that the UT urban PM_2.5_ problem is acute. With the increasing population in northern UT, air quality standards will likely not be attainable without greater use of the public train systems which have been installed over the last decade or so with associated incentives to ride and disincentives for driving. Other examples of upstream policies relevant to asthma action plans and PM_2.5_ pollution with potential for widespread change include: The 2014 UT Indoor Air Act, banning smoking in public areas; land-use policies in desert/arid areas aimed at limiting and controlling airborne dust near centers of population [80]; reducing costly AER visits (Healthy People 2020 Objective RD-3) [1] by removing barriers to routine asthma care by ensuring that health providers are reimbursed by insurance companies for one-on-one patient education sessions [1]; expanding the UT Clean Fuels Programs (the UT Clean Fuels Programs provide grants, loans and tax credits to businesses and individuals for electric vehicle and clean fuel use) [13] in PM_2.5_ non-attainment areas; expanding monitoring of PM_2.5_ by installing more PM_2.5_ monitoring stations and monitoring PM_10_ especially in rural UT. Additionally, the use of community databases of socio-economic factors as described by Novilla et al. [86] and the UT HII [55] may provide the key to lowering the social gradient by reducing USAD level inequalities and getting SDH on the public health policy map in the state of UT. USAD level studies such as the HII index concentrating on northern and southern UT separately are needed to detect changes in factors at the community level. Addressing these issues will also provide a platform for alignment with the integrative approach of the new sustainable development goals [11] and may expose regional weaknesses in the healthcare system building blocks, especially responsiveness [87].

## 5. Conclusions

The present study demonstrated the association of several factors with asthma and revealed inequalities in the state of UT. Counties with the highest AAP and AER rates had high amounts of mining activities, wind erosion risk and lower socio-economic status. Counties with more moderate rates of AAP and AER visits were located in the densely populated north of the state where traffic pollution drives PM_2.5_ levels. Examination of the links between socio-economic (HII) and PM_2.5_ data at the USAD level showed large disparities within northern counties with the least affluent USAD areas having higher pollution levels and higher AAP. The clear differences in socio-economic characteristics and causes of pollution between the north and south of the state warrant separate study for smaller geographical areas within these regions. Action to tackle asthma exacerbation needs to be multifaceted and multi-sectoral, prioritizing resources and programs in rural UT counties experiencing the multiple burdens of aridity/desert/mining dust and reduced access to healthcare due to proximity and lower socio-economic status. Inequalities in smoking should inform targeting of local reduction campaigns. Future research using spatial analysis is needed in UT to build on the evidence base thus far and inform prioritization of scarce resources. Proper monitoring of desert and mining dust is needed. This could be achieved through installing monitoring stations in mid-sized towns in southern UT or analysis and calibration of satellite imagery, particularly during winter temperature inversions and summer dust storm events. Individual level studies in northern and southern UT that investigate ER visit dates and the weather conditions that could influence pollution levels on those dates are also needed. As well as ER visits, family doctor visits should be considered to gain a more complete understanding of the number of cases. As more small area data become available state-wide, higher spatial resolution trends could be modeled.

## Figures and Tables

**Table 2:** Mean ranks of risk factor variables for low and high AAP groups (Mann-Whitney U test) and low, medium and high AAP groups (Kruskal-Wallis H test) and Pearson correlation of risk factors with AAP.

	Mann-Whitney U Test			Kruskal-Wallis H Test				Pearson Correlation with AAP	*p*-Value (2 dp)
	Mean Rank		*p*-Value	Mean Rank			*p*-Value (2 dp)		
Risk Factor Variables	Low AAP	High AAP		Low AAP	Medium AAP	High AAP			
**Environmental variables**									
Estimated Mine Area	12.86	20.63	0.03 *	7.50	14.53	20.63	0.02 *	0.45	0.01 **
Median Aridity Index	17.43	8.63	0.01 **	20.80	16.38	8.63	0.03 *	−0.48	0.01 **
Minimum Aridity Index	16.90	10.00	0.05 *	19.00	16.25	10.00	0.12	−0.35	0.06 §
Median Elevation (m)	14.86	15.38	0.88	21.80	12.69	15.38	0.11	−0.30	0.12
CL PM_2.5_	15.86	12.75	0.40	19.40	14.75	12.75	0.38	−0.06	0.77
Red Air Days	17.02	9.69	0.03 *	18.20	16.66	9.69	0.09 §	−0.24	0.22
Total Mines	12.95	20.38	0.02 *	8.00	14.50	20.38	0.02 *	0.33	0.08 §
Wind Erosion Risk	12.83	20.69	0.02 *	7.00	14.72	20.56	0.02 *	0.45	0.01 **
**Health behavior variables**									
Obesity (%)	13.36	19.31	0.09 §	13.90	13.19	19.31	0.24	0.49	0.01 **
Smoking (%)	13.48	19.00	0.11	9.10	14.84	19.00	0.12	0.42	0.02 *
**Socio-economic variables**									
Health Improvement Index	13.76	18.25	0.21	3.8	16.88	18.25	0.005 **	0.49	0.01 **
Median Household Income	16.90	10.00	0.05 *	20.60	15.75	10.00	0.08 §	−0.30	0.11
Native American Pop.	14.24	17.00	0.37	12.90	14.66	17.00	0.60	0.14	0.47
Population Density	17.02	9.69	0.04 *	15.40	7.53	9.69	0.10	−0.11	0.56
Poverty (%)	12.43	21.75	0.01 **	7.60	13.94	21.75	0.01 **	0.35	0.06 §
Unemployment (%)	13.38	19.25	0.10 §	14.80	12.94	19.25	0.23	0.14	0.47
Uninsured (%)	13.64	18.56	0.16	11.50	14.31	18.56	0.30	0.09	0.66

§ significant at *p* = 0.1, * significant at *p* = 0.05, ** significant at *p* = 0.01.

**Table 3:** Regression results for AAP (R^2^ = 0.425).

Term	Parameter Estimates	Standard Error	*p*-Value
Intercept	7.03237	2.30619	0.006 *
Median AI	−0.00042	0.00022	0.066
Total Mines	0.14935	0.09469	0.128
Smoking	0.32907	0.20596	0.123
Native Am. Pop.	−0.00009	0.00004	0.028 *

* Significant at *p* < 0.05.

**Table 4:** Correlations Between RK AAP and CT PM_2.5_ data for metro and non-metro small areas.

			Pearson Correlations with RK AAP			
Variable	All Small Areas	*p*-Value (n = 588)	Metro Small Areas	*p*-Value (n = 519)	Non-Metro Small Areas	*p*-Value (n = 69)
CT PM_2.5_ Summer 2011 max	0.273	<0.01 **	0.396	<0.01 **	0.211	0.08 §
CT PM_2.5_ Summer 2014 max	0.022	0.59	0.378	<0.01 **	−0.161	0.19
CT PM_2.5_ Winter 2011–2014 mean	0.090	0.03 *	0.428	<0.01 **	−0.06	0.62

§ significant at *p* = 0.1, * significant at *p* = 0.05, ** significant at *p* = 0.01.

**Table 5:** Mean ranks of risk factor variables for low and high AER groups (Mann-Whitney U test) and low, medium and high AER groups (Kruskal-Wallis H test) and Pearson correlation of risk factors with AER visits.

	Mann-Whitney U Test			Kruskal-Wallis H Test				Pearson Correlation with AER Visits	*p*-Value (2 dp)
	Mean Rank		*p*-Value	Mean Rank			*p*-Value (2 dp)		
	Low AER	High AER		Low AER	Medium AER	High AER			
**Environmental variables**									
Estimated Mine Area	14.35	15.53	0.71	14.58	12.38	21.36	0.06 §	0.25	0.19
Median Aridity Index	17.62	12.88	0.14	16.00	17.38	8.71	0.08 §	−0.38	0.04*
Minimum Aridity Index	17.27	13.16	0.20	14.00	18.69	7.43	0.01 **	−0.38	0.04 *
Median Elevation (m)	15.77	14.38	0.66	19.33	14.31	12.86	0.35	−0.23	0.23
CL PM_2.5_	16.46	13.81	0.40	15.75	17.56	8.51	0.06 §	−0.33	0.08 §
Red Air Days	18.12	12.47	0.07 §	14.25	18.28	8.14	0.02 *	−0.31	0.11
Total Mines	14.73	15.22	0.87	16.83	12.31	19.57	0.10 §	0.25	0.19
Wind Erosion Risk	12.75	11.03	0.59	16.25	11.91	21.00	0.05 *	0.51	0.01 **
**Health behavior variables**									
Obesity (%)	15.96	14.22	0.58	18.25	13.59	15.43	0.51	0.16	0.42
Smoking (%)	16.23	14.00	0.48	20.17	11.31	19.00	0.03 *	0.02	0.93
**Socio-economic variables**									
Health Improvement Index	15.14	14.87	0.95	15.00	13.88	17.57	0.63	0.13	0.50
Median Household Income	16.38	13.88	0.43	13.17	18.06	9.57	0.08 §	−0.17	0.38
Native American Pop.	13.69	16.06	0.39	14.92	13.22	19.14	0.21	0.37	0.05 *
Population Density	17.23	13.19	0.20	12.33	17.97	10.50	0.11	−0.21	0.28
Poverty (%)	15.81	14.34	0.65	17.92	12.09	19.14	0.12	0.15	0.43
Unemployment (%)	14.54	15.38	0.79	18.50	12.69	17.29	0.26	0.15	0.45
Uninsured (%)	12.65	16.91	0.18	13.92	12.81	20.93	0.10 §	0.37	0.05 *

§ significant at *p* = 0.1, * significant at *p* = 0.05, ** significant at *p* = 0.01.

**Table 6:** Regression Results for AER visits (R^2^ = 0.307).

Term	Parameter Estimates	Standard Error	*p*-Value
Intercept	15.7536	6.0839	0.0155 *
Wind Erosion Risk	1.0798	0.4307	0.0187 *
Uninsured	0.4142	0.3088	0.1914

* Significant at *p* < 0.05.

**Table 7:** Summary of all associations between risk factors and AAP found in statistical analysis (Red indicates positive association, blue indicates negative association, yellow indicates changing association).

AAP (County Level)	Expected Association	Mann Whitney U	Kruskal Wallis H	Pearson Correlation	Regression
Risk Factor Variables	Positive or Negative	Ranks Associated with High AAP (HL)	Ranks Associated with High AAP (and Low AAP) (HML)	Positive or Negative	Positive or Negative
**Environmental variables**					
Estimated Mine Area	pos.	H *	H(L) *	pos. **	-
Median Aridity Index	neg.	L **	L(H) *	neg.**	neg. §
Minimum Aridity Index	neg.	L *	L(H)	neg. §	-
Median Elevation (m)	neg.	H	M(H)	neg.	-
CL PM_2.5_	pos.	L	L(H)	neg.	-
Red Air Days	pos.	L *	L(H) §	neg.	-
Total Mines	pos.	H *	H(L) *	pos. §	pos. §
Wind Erosion Risk	pos.	H *	H(L) *	pos. **	-
**Health behavior variables**					
Obesity (%)	pos.	H §	H(M)	pos. **	-
Smoking (%)	pos.	H	H(L).	pos. *	pos. §
**Socio-economic variables**					
Health Improvement Index	pos.	H	H(L) **	pos. **	-
Median Household Income	neg.	L *	L(H) §	neg.	-
Native American Pop.	pos.	H	H(L)	pos.	neg *
Population Density	pos.	L *	M(H)	neg.	-
Poverty (%)	pos.	H *	H(L) **	pos. §	-
Unemployment (%)	pos.	H §	H(M)	pos.	-
Uninsured (%)	pos.	H	H(L).	pos.	-
	**Correlations for Metro vs. Non-Metro**				
**RK AAP (USAD)**	**Expected Association**	**All**	**Metro**	**Non-Metro**	
CT PM_2.5_ Summer 2011 max.	pos.	pos. **	pos. **	pos. §	
CT PM_2.5_ Summer 2014 max.	pos.	pos.	pos. **	neg.	
CT PM_2.5_ Winter 2011-2014 mean	pos.	pos. *	pos. **	neg.	
	**Moving Correlations with RK AAP**				
**RK AAP (USAD)**		**Northern UT**	**Southern UT**		
Population Density	pos.	pos.	neg.		
CT PM_2.5_ Summer 2011 max.	pos.	pos.	neg.		
CT PM_2.5_ Winter 2011-2014 mean	pos.	pos.	neg.		

§ significant at *p* = 0.1, * significant at *p* = 0.05, ** significant at *p* = 0.01.

**Table 8:** Summary of all associations between risk factors and AER found in statistical analysis (red indicates positive association, blue indicates negative association, yellow indicates changing association).

AER (County Level)	Expected Association	Mann Whitney U		Kruskal Wallis H		Pearson Correlation	Regression
Risk Factor Variables	Positive or Negative	Ranks Associated with High AER (HL)		Ranks Associated with High AER (HML)		Positive or Negative	Positive or Negative
**Environmental variables**							
Estimated Mine Area	pos.	H		H(M) §		pos.	-
Median Aridity Index	neg.	L		L(M) §		neg. *	-
Minimum Aridity Index	neg.	L		L(M) **		neg. *	-
Median Elevation (m)	neg.	L		L(H)		neg.	-
CL PM_2.5_	pos.	L		L(M) §		neg. §	-
Red Air Days	pos.	L §		L(M) *		neg.	-
Total Mines	pos.	H.		H(M) *		pos.	-
Wind Erosion Risk	pos.	L		H(M) *		pos. **	pos. *
**Health behavior variables**							
Obesity (%)	pos.	L		M(H)		pos.	-
Smoking (%)	pos.	L		M(H) *		pos.	-
**Socio-economic variables**							
Health Improvement Index	pos.	L		H(M)		pos.	-
Median Household Income	neg.	L		L(M) §		neg.	-
Native American Pop.	pos.	H.		H(M)		pos. *	-
Population Density	pos.	L		L(M)		neg.	-
Poverty (%)	pos.	L		H(M)		pos.	-
Unemployment (%)	pos.	H.		M(H)		pos.	-
Uninsured (%)	pos.	H.		H(M) §		pos. *	pos.
	**Moving Correlations with RK AER**						
**RK AER (USAD)**	**Expected Association**	**Northern UT**		**Southern UT**			
HII	pos.	pos.	neg.	pos.	neg.		
Population Density	pos.	pos.	neg.	pos.	neg.		
Obesity	pos.	pos.	neg.	pos.	neg.		

§ significant at *p* = 0.1, * significant at *p* = 0.05, ** significant at *p* = 0.01.

## Supplementary Materials

The following are available online at https://www.mdpi.com/1660-4601/17/14/5251/s1.

## References

[1] UT Department of Health UDOH (2012). Asthma in UT Burden Report 2012. http://health.UT.gov/asthma/data/reports/burdenreport/burdenreport2012.pdf.

[2] Centers for Disease Control and Prevention CDC Most Recent Asthma Data. National Health Interview Survey NHIS. https://www.cdc.gov/asthma/most_recent_data.htm.

[3] National Ambulatory Medical Care Survey NAMCS National Ambulatory Medical Care Survey 2012. www.cdc.gov/nchs/data/ahcd/namcs_web_tables.pdf.

[4] Hasegawa K., Bittner J., Nonas S., Stoll S., Watase T., Gabriel S., Herrera V., Camargo C. (2015). Children and adults with frequent hospitalizations for asthma exacerbation 2012–2013: A multicenter observational study. Am. Acad. Allergy Asthma Immunol..

[5] Asthma and Allergy Foundation of America Cost of Asthma on Society. http://www.aafa.org/page/cost-of-asthma-on-society.aspx.

[6] Halldin C., Doney B., Hnizdo E. (2015). Changes in prevalence of chronic obstructive pulmonary disease and asthma in the US population and associated risk factors. Chron. Resp. Dis..

[7] (2012). American Lung Association ALA Trends in Asthma Morbidity and Mortality. www.lung.org/finding-cures/our-research/trend-reports/asthma-trend-report.pdf.

[8] (2016). UT Department of Health UDOH Asthma Fast Stats. http://health.UT.gov/asthma/fast/stats.

[9] Toskala E., Kennedy D. (2015). Asthma risk factors. Int. Forum Allergy Rhinol..

[10] World Health Organization WHO (2016). Ambient Air Pollution: A Global Assessment of Exposure and Burden of Disease. https://apps.who.int/iris/bitstream/handle/10665/250141/9789241511353-eng.pdf.

[11] (2015). United Nations UN Sustainable Development Goals. https://sustainabledevelopment.un.org/?menu=1300.

[12] Burnett R., Chen H., Szyszkowicz M., Fann N., Hubbell B., Pope C.A., Apte J.S., Brauer M., Cohen A., Weichenthal S. (2018). Global estimates of mortality associated with long-term exposure to outdoor fine particulate matter. Proc. Natl. Acad. Sci. USA.

[13] (2016). Environmental Protection Agency EPA Criteria Air Pollutants. https://www.epa.gov/criteria-air-pollutants.

[14] Cox L., Popken D. (2015). Has reducing fine particulate matter and ozone caused reduced mortality rates in the United States?. Ann. Epidemiol..

[15] University of Southern California USC (2016). ‘Southern California’s reduction in smog linked to major improvement in children’s health: Bronchitic symptoms on the decline as pollution levels drop in Los Angeles region over the past two decades. Sci. News.

[16] Bon R.L., Krahulec K.A. (2009). Summary of Mineral Activity in UT. Circular 111 UT Geological Survey.

[17] National Weather Service Forecasting Office (2016). What are Temperature Inversions?. http://www.wrh.noaa.gov/slc/climate/TemperatureInversions.php.

[18] American Lung Association (2016). State of the Air Report 2016: Most Polluted Cities. www.lung.org/our-initiatives/healthy-air/sota/city-rankings/most-polluted-cities.html.

[19] American Lung Association (2019). State of the Air 20th Anniversary Report 2019. https://www.lung.org/assets/documents/healthy-air/state-of-the-air/sota-2019-full.pdf.

[20] Whiteman D., Hoch S., Horel J., Charland A. (2014). Relationship between particulate air pollution and meteorological variables in UT’s Salt Lake Valley. Atmo. Environ..

[21] Malek E., Davis T., Martin R., Silva P. (2006). Meteorological and environmental aspects of one of the worst national air pollution episodes (January 2004) in Logan, Cache Valley, UT, USA. Atmos. Res..

[22] Johnson R., Graham G. (2005). Fine particulate matter National Ambient Air Quality Standards: Public health impact on populations in the Northeastern United States. Environ. Health Perspect..

[23] Esworthy R. (2015). Air Quality: EPA’s 2013 Changes to the Particulate Matter (PM) Standard. Congressional Research Service Report.

[24] Di Q., Dai L., Wang Y., Zanobetti A., Choirat C., Schwartz J.D., Dominici F. (2017). Association of Short-term exposure to air pollution with mortality in older adults. JAMA.

[25] Robert Wood Johnson Foundation RWJF (2016). County Health Rankings. http://www.countyhealthrankings.org.

[26] Alvarez B., Echeverria S., Alvarez S., Krupa S. (2013). Air quality standards for particulate matter (PM) at high altitude cities. Environ. Pollut..

[27] Johnson K. (2011). Quality of Air? That’s as Murky as Western Sky. New York Times.

[28] Goodman M.M., Carling G.T., Fernandez D.P., Rey K., Hale C., Bickmore B., Nelson S., Munroe J. (2019). Trace element chemistry of atmospheric deposition along the Wasatch Front (UT, USA) reflects regional playa dust and local urban aerosols. Chem. Geol..

[29] Anto J. (2012). Recent advances in the epidemiologic investigation of risk factors for asthma: A review of the 2011 literature. Curr. Allergy Asthma Rep..

[30] Mirabelli M., Vaidyanathan A., Flanders W., Qin X., Garbe P. (2016). Outdoor PM2.5, Ambient Air Temperature, and Asthma Symptoms in the past 14 Days among Adults with Active Asthma. Environ. Health Perspect..

[31] Akinbami L., Lynch C., Parker J., Woodruff T. (2010). The association between childhood asthma prevalence and monitored air pollutants in metropolitan areas, United States 2001–2004. Environ. Res..

[32] Peel J., Tolbert P., Klein M., Metzger K., Flanders W., Todd K., Mulholland J., Ryan P., Frumkin H. (2005). Ambient air pollution and respiratory emergency visits. Epidemiology.

[33] Gorai A., Tchounwou P., Tuluri F. (2016). Association between ambient air pollution and asthma prevalence in different population groups residing in eastern Texas, USA. Int. J. Environ. Res. Public Health.

[34] Beard J., Beck C., Graham R., Packham S., Traphagan M., Giles R., Morgan J. (2012). Winter temperature inversions and emergency department visits for asthma in Salt Lake County, UT, 2003—2008. Environ. Health Perspect..

[35] Lauer F., Mitchell L., Bedrick E., McDonald J., Lee W., Li W., Olvera H., Amaya M., Berwick M., Gonzales M. (2009). Temporal-spatial analysis of U.S.-Mexico border environmental fine and coarse PM air sample extract activity in human bronchial epithelial cells. Toxicol. Appl. Pharm..

[36] Wallace J., Nair P., Kanaroglou P. (2010). Atmospheric remote sensing to detect effects of temperature inversions on sputum cell counts in airway diseases. Environ. Res..

[37] Thalib L., Al-Taiar A. (2012). Dust storms and the risk of asthma admissions to hospitals in Kuwait. Sci. Total Environ..

[38] Watanabe M., Yamasaki A., Burioka N., Kurai J., Yoneda K., Yoshida A., Igishi T., Fukuoka Y., Nakamoto M., Takeuchi H. (2011). Correlation between Asian Dust Storms worsening asthma in Western Japan. Allergol. Int..

[39] Strannegard I., Strannegard O. (1990). Childhood bronchial asthma in a desert county. Allergy.

[40] Akpinar-Elci M., Martin F., Behr J., Diaz R. (2015). Saharan dust, climate variability, and asthma in Grenada, the Caribbean. Int. J. Biotechnol..

[41] Kiechl-Kohlendorfer U., Horak E., Mueller W., Strobl R., Haberland C., Fink F., Schwaiger M., Gutenberger K., Reich H., Meraner D. (2007). Living at high altitude and risk of hospitalisation for atopic asthma in children: Results from a large prospective birth-cohort study. Arch. Dis. Child..

[42] Cogo A., Fiorenzano G. (2009). Bronchial asthma: Advice for patients traveling to high altitude. High Alt. Med. Biol..

[43] Vargas M.H., Becerril-Angeles M., Medina-Reyes I.S., Rascon-Pacheco R.A. (2018). Altitude above 1500 m is a major determinant of asthma incidence: An ecological study. Respir. Med..

[44] Ayaaba E., Li Y., Yuan J.L., Ni C.H. (2017). Occupational Respiratory Diseases of Miners from Two Gold Mines in Ghana. Int. J. Environ. Res. Public Health.

[45] Azad S. (2015). Environmental Degradation due to Coal Mining in Baluchistan. Pol. J. Environ. Stud..

[46] Neophytou A.M., Costello S., Picciotto S., Noth E.M., Liu S., Lutzker L., Balmes J.R., Hammond K., Cullen M.R., Eisen E.A. (2019). Accelerated lung function decline in an aluminium manufacturing industry cohort exposed to PM2.5: An application of the parametric g-formula. Occup. Environ. Med..

[47] Dunway M.C., Pfennigwerth A.A., Fick S., Nauman T.W., Belnap J., Barger N. (2019). Wind erosion and dust from US drylands: A review of causes, consequences, and solutions in a changing world. Ecosphere.

[48] Khokhawalla S., Rosenthal S., Pearlman D., Triche E. (2015). Cigarette smoking and emergency care utilization among asthmatic adults in the 2011 Asthma Call-back Survey. J. Asthma Off. J. Assoc. Care Asthma.

[49] Cerveri I., Cazzoletti L., Corsico A., Marcon A., Niniano R., Grosso A., Ronzoni V., Accordini S., Janson C., Pin I. (2012). The impact of cigarette smoking on asthma: A population-based international cohort study. Int. Arch. Allergy Immunol..

[50] Centers for Disease Control and Prevention (2010). Asthma and Obesity. https://www.cdc.gov/asthma/asthma_stats/asthma_obesity.htm.

[51] Akerman M.J., Calacanis C.M., Madsen M.K. (2004). Relationship between asthma severity and obesity. J. Asthma.

[52] Jackson E., Doescher M., Hart L. (2007). National Study of Lifetime Asthma Prevalence and Trends in Metro and Non-Metro Counties, 2000–2003.

[53] UT Department of Environmental Quality UDEQ (2014). UT Division of Air Quality 2014 Annual Report. http://www.airquality.UT.gov/docs/2015/02Feb/2014DAQAnnualReport_FINAL.pdf.

[54] Berkman L.S. (2009). Social Epidemiology: Social Determinants of Health in the United States: Are We Losing Ground?. Annu. Rev. Public Health.

[55] UT Department of Health (2018). The UT Health Improvement Index. https://health.UT.gov/disparities/data/ohd/UTHII.pdf.

[56] Centers for Disease Control and Prevention (2012). Adult Asthma Data: Prevalence Tables and Maps. https://www.cdc.gov/asthma/brfss/2012/tablel7.htm.

[57] Grineski S., Staniswalis J., Bulatgsinhala P., Peng Y., Gill T. (2011). Hospital admissions for asthma and acute bronchitis in El Paso, Texas: Do age, sex, and insurance status modify the effects of dust and low wind events?. Environ. Res..

[58] Kim S., O’Neill M., Lee J., Cho Y., Kim J., Kim H. (2007). Air pollution, socioeconomic position, and emergency hospital visits for asthma in Seoul, Korea. Int. Arch. Occup. Environ. Health.

[59] Akinbami L.J., Moorman J.E., Bailey C. (2012). Trends in Asthma Prevalence, Health Care Use, Mortality in the United States 2001–2010.

[60] Forno E., Celedon J.C. (2009). Asthma and ethnic minorities: Socioeconomic status and beyond. Curr. Opin. Allergy Clin. Immunol..

[61] Gorman B.K., Chu M. (2009). Racial and ethnic differences in adult asthma prevalence, problems, and medical care. Ethn. Health.

[62] Coggon D., Rose G., Barker D. (2003). Epidemiology for the Uninitiated.

[63] Loney T., Nagelkerke N. (2014). The individualistic fallacy, ecological studies and instrumental variables: A causal association. Emerg. Themes Epidemiol..

[64] United States Census Bureau USCB (2016). UT is nation’s fastest-growing state, Census Bureau reports. http://www.census.gov/newsroom/press-releases/2016/cb16-214.html.

[65] UT Department of Health UDOH (2016). Health Indicator Report of Asthma: Adult Prevalence. https://ibis.health.UT.gov/indicator/view/AsthAdltPrev.SA.html.

[66] (2016). Health Indicators Warehouse HIW. http://www.healthindicators.gov.

[67] Centers for Disease Control and Prevention CDC Emergency Department Visits for Asthma: Age-Adjusted Rate of Emergency Department Visits for Asthma per 10,000 Population. Natl. Environ. Public Health Track. Netw..

[68] Vaidyanathan A., Dimmick W.D., Kegler S.R., Qualters J.R. (2013). Statistical Air Quality Predictions for Public Health Surveillance: Evaluation and Generation of County-level Metrics of PM2.5 for the Environmental Public Health Tracking Network. Int. J. Health Geogr..

[69] Berrocal V., Gelfand A.E., Holland D.M. (2011). Space-time fusion under error in computer model. Biometrics.

[70] (2011). Output: An application to modeling air quality. Biometrics.

[71] Berrocal V., Gelfand A.E., Holland D.M. (2010). A bivariate space-time downscaler under space and time misalignment. Ann. Appl. Stat..

[72] Berrocal V., Gelfand A.E., Holland D.M. (2010). A spatio-temporal downscaler for output from numerical models. J. Agric. Biol. Environ. Stat..

[73] (2005). United States Geological Survey USGS. http://egsc.usgs.gov/lsb/pubs/booklets/elvadlst.html.

[74] Global Aridity and PET Database. www.cgjar-csi.org/data/global-aridity-and-pet-database.

[75] Krahulec K. (2008). UT Mining Districts.

[76] Jacquez G.M., Goovaerts P., Kaufmann A., Rommel R. (2014). SpaceStat 4.0 User Manual: Software for the Space-Time Analysis of Dynamic Complex Systems, 04/2014.

[77] Hengl T., Heuvelink G.B., Rossiter D.G. (2007). About regression-kriging: From equations. Comput. Geosci..

[78] Aneslin L. (1995). Local Indicators of Spatial Association--LISA. Geog. Anal..

[79] Goovaerts P., Jacquez G.M. (2005). Detection of temporal changes in the spatial distribution of cancer rates using local Maran’s I and geostatistically simulated spatial neutral models. J. Geog. Syst..

[80] Belnap J., Walker B., Munson S., Gill R. (2014). Controls on sediment production in two U.S. deserts. Aeolian Res..

[81] Wilhelm M., Ritz B. (2005). Local Variations in CO and Particulate Air Pollution and Adverse Birth Outcomes in Los Angeles County, California, USA. Environ. Health. Perspect..

[82] Merrill R. (2004). Descriptive Findings Life Expectancy among LDS and Non-LDS in UT. Demogr. Res..

[83] Schultze-Weminghause G. (2008). Effects of high altitude on bronchial asthma. Pneumologie.

[84] Simeonov K., Himmelstein D. (2015). Lung cancer incidence decreases with elevation: Evidence for oxygen as an inhaled carcinogen. Peer J..

[85] Wilkinson R., Marmot M., World Health Organization Social Determinants of Health.

[86] Novilla L., Barnes M., Hanson C., West J., Edwards E. (2011). How Can We Get the Social Determinants of Health Message on the Public Policy and Public Health Agenda?. www.who.int/sdhconference/resources/draft_background_paper2_usa.pdf.

[87] World Health Organization WHO The WHO Health Systems Framework. https://www.who.int/healthsystems/strategy/everybodys_business.pdf.

