# Supplementary material for: Investigation of the Environmental and Socio-Economic Characteristics of Counties with a High Asthma Burden to Focus Asthma Action in Utah
Source: Int J Environ Res Public Health. 2020 Jul 21;17(14):5251. doi: 10.3390/ijerph17145251 (PMC7400464; doi:10.3390/ijerph17145251)
Supplement: Supplementary file 1 [file ijerph-17-05251-s001.pdf]

International Journal of
Environmental Research and Public Health

MDPI

Supplementary Materials

Investigation of the Environmental and Socio-Economic Characteristics of Counties with a High Asthma Burden to Focus Asthma Action in Utah

Maureen Vowles [1], Ruth Kerry [2], Ben Ingram [3] and Linda Mason [4]

[1] Department of Public Health and Policy, University of Liverpool, Liverpool, L69 3GB, UK; mmvowles@gmail.com
[2] Department of Geography, Brigham Young University, Utah, 84602, USA; ruth_kerry@byu.edu
[3] Faculty of Engineering, Universidad de Talca, Curicó, 3460000, Chile; ingrambr@gmail.com
[4] Liverpool School of Tropical Medicine, Liverpool, L3 5QA, UK; Linda.Mason@lstmed.ac.uk
* Correspondence: mmvowles@gmail.com

1. Abbreviation Guide

AAP	Adult Asthma Prevalence
AER	Asthma Emergency Room
AI	Aridity Index
BRFSS	US Behavioral Risk Factor Surveillance System
CAA	Clean Air Act
CDC	Centers for Disease Control and Prevention
CL $PM_{2.5}$	County Level Particulate Matter with Diameter less than 2.5 micrometers
CT $PM_{2.5}$	Census Tract Level Particulate Matter with diameter less than 2.5 micrometers
DEM	Digital Elevation Model
EPA	Environmental Protection Agency
EPHT	Environmental Public Health Tracking Network
ER	Emergency Room
HII	Health Improvement Index
HH	High value surrounded by high values
HP-2020	Health People 2020
HL	High value surrounded by low values
KSL	K Salt Lake Radio/TV network
LH	Low value surrounded by high values
LL	Low value surrounded by low values
LMI	Local Moran's I
MAE	Mean Annual Potential Evapotranspiration
MAP	Mean Annual Precipitation
MHHI	Median Household Income
MLR	Multiple Linear Regression
NAAQS	National Ambient Air Quality Standards
NHIS	National Health Interview Survey
$PM_{2.5}$	Particulate Matter with diameter less than 2.5 micrometers)
PM_{10}	Particulate Matter with diameter less than 10 micrometers)
RK	Regression Kriging
RMSE	Root Meat Square Error
SAHIE	Small Area Health Insurance Estimates
SDH	Social Determinants of Health
SRTM	Shuttle Radar Topography Model
USAD	Utah Small Area Data

USGS	United States Geological Survey
UT	Utah
WHO	World Health Organization

2. Methods

2.1. Questions used from BRFSS to determine AAP, smoking and obesity

AAP, smoking and obesity rates were determined from the BRFSS using the following questions:

1)"Have you ever been told by a doctor {nurse or other health professional} that you have asthma?" Current asthma is defined as an affirmative response to that question followed by an affirmative response to the subsequent question "Do you still have asthma?" [65] and 2) "Do you now smoke cigarettes every day, some days, or not at all?" [66], with individuals reporting any smoking being considered as smokers. Obesity was determined from BRFSS questions about height and weight and calculated based on a BMI of 30-99.8 [66].

2.2. CL PM2.5 Data limitations

The following statement of data limitations comes with the CL PM2.5 data: "measures estimate average annual concentration of fine PM pollution in the county, and can miss "important short-term fluctuations in air quality (such as stagnation events), local patterns (high concentrations near roads and other major sources), and other pollutants (such as ozone, etc.). Further, these estimates are based on seasonal averages. Even within counties with low average fine PM concentrations, locations can experience days of dangerously elevated levels. It should be noted that these data are derived from only one air quality model among several. Like all models, this air quality model has errors. There is also a large time lag (up to 5 years) between when these data are collected and when the modeled results become available." https://www.countyhealthrankings.org/app/UT/2019/measure/factors/125/description

2.3. Socio-economic indicators used in HII calculation

1) adults (over 25) with <9 years education, 2) Adults (over 25) with at least a high school diploma, 3) median family income, 4) income disparity, 5) owner occupied houses, 6) unemployment, 7) families below poverty level, 8) population below 150% of poverty threshold and 9) single parent households with children.

3. Results

3.1. Classification of Utah Counties as Metro (M) and Non-metro (N)

Beaver	N
Box Elder	M
Cache	M
Carbon	M
Daggett	N
Davis	M
Duchesne	N
Emery	N
Garfield	N
Grand	N
Iron	M
Juab	M
Kane	N
Millard	N
Morgan	M
Piute	N
Rich	N
Salt Lake	M
San Juan	N
Sanpete	N
Sevier	N
Summit	M
Tooele	M
Uintah	M
Utah	M
Wasatch	M
Washington	M
Wayne	N
Weber	M

(M = Metropolitan/Micropolitan counties; N = Non-metropolitan counties. Source: USDA (2013))

United States Department of Agriculture USDA (2013) 'What is rural?' [Online] Available from: https://www.ers.usda.gov/topics/rural-economy-population/rural-classifications/what-is-rural.aspx [Accessed: 3 March 2017]

Table 1. Correlation matrix to Pearson coefficients for all counties

	Population Density	Native American Population	Estimated Mine Area	Median AI	Min. AI	AER	AAP	PM2.5	Red Air Days	Elevation	Uninsured	Smoking	Wind Erosion Risk	Poverty	MHHI	Obesity	Unemployment	Total Mines	HII
Population Density	1	-0.098	-0.169	0.264	.455*	-0.136	0.010	0.276	.748**	-.384*	-0.252	-0.132	-0.165	-0.189	0.309	-0.111	-0.195	-0.128	-0.016
Native American Population		1	0.312	-0.232	-0.291	0.287	0.071	-.410*	-0.188	-0.109	.452*	.683**	0.314	.760**	-0.258	0.180	.608**	.832**	.389*
Estimated Mine Area			1	-.533**	-.469*	0.250	.450*	-0.238	-0.269	-0.270	0.142	.434*	.999**	.395*	-0.258	0.266	0.295	.649**	.498**
Median AI				1	.894**	-.381*	-.479**	0.319	.631**	.501**	-0.358	-.401*	-.524**	-.387*	.602**	-.564**	-.425*	-.382*	-.525**
Min. AI					1	-.378*	-0.350	.433*	.753**	0.241	-.464*	-.422*	-.456*	-.418*	.610**	-.416*	-.550**	-.422*	-.433*
AER						1	0.045	-0.327	-0.307	-0.231	.374*	0.017	0.509	0.153	-0.170	-0.114	0.145	0.252	0.130
AAP							1	-0.057	-0.237	-0.298	0.086	.418*	.453*	0.354	-0.302	.370*	0.139	0.329	.492**
PM2.5								1	.412*	-0.065	-.478**	-0.340	-0.214	-.472**	.508**	0.103	-.586**	-.460*	-.369*
Red Air Days									1	-0.243	-.486**	-0.324	-0.261	-.388*	.631**	-0.320	-0.365	-0.267	-0.306
Elevation										1	0.055	-0.133	-0.266	-0.051	0.002	-0.353	-0.089	-0.142	-0.359
Uninsured											1	.439*	0.142	.640**	-.708**	0.095	.423*	0.313	.607**
Smoking												1	.439*	.756**	-.490**	.383*	.640**	.728**	.585**
Wind Erosion Risk													1	.398*	-0.251	0.269	0.291	.651**	.493**
Poverty														1	-.685**	0.218	.535**	.748**	.664**
MHHI															1	-0.325	-.451*	-0.326	-.658**
Obesity																1	0.103	0.234	0.312
Unemployment																	1	.690**	.396*
Total Mines																		1	.470*
HII																			1

*Correlation is significant at p=0.05 **Correlation is significant at p=0.01 level

Table 2 Pearson Correlation Matrix for Metro Counties

	Pop. Density	Native American Populattion	Estimated Mine Area	Median AI	Min.AI	AER	AAP	PM2.5	Red Air Days	Elevation	Uninsured	Smoking	Poverty	MHHI	Obesity	Unemploy ment	Total Mines	HII
Pop.density	1	-0.145	-0.229	0.126	0.378	-0.102	0.056	0.288	.735**	-0.404	-0.131	-0.036	-0.140	0.138	-0.024	-0.124	-0.158	0.139
Native American		1	0.284	-0.317	-0.276	.542*	0.076	0.127	-0.347	-0.014	0.311	0.208	0.082	-0.061	0.315	-0.262	0.011	0.005
Estimated Mine			1	-.588*	-.536*	-0.082	.561*	0.304	-0.380	-0.134	-0.009	0.387	0.368	-0.425	0.482	0.152	.821**	.573*
Median AI				1	.876**	-0.182	-.558*	-0.173	.517*	.674**	-0.124	-0.384	-0.377	.522*	-.724**	-0.253	-0.433	-0.488
Min.AI					1	-0.269	-0.425	-0.073	.670**	0.387	-0.268	-0.334	-0.341	0.475	-0.499	-0.398	-0.486	-0.295
AER						1	-0.338	-0.282	-0.304	-0.091	0.408	-0.310	-0.220	0.088	-0.109	-0.437	-0.368	-0.174
AAP							1	0.250	-0.271	-0.363	-0.144	.672**	0.434	-0.444	.533*	.531*	.695**	.562*
PM2.5								1	0.238	-0.236	-0.408	0.116	-0.399	0.408	0.391	0.348	0.227	0.042
Red Air Days									1	-0.156	-0.343	-0.242	-0.396	0.422	-0.226	-0.192	-0.383	-0.222
Elevation										1	0.101	-0.218	-0.034	0.246	-.691**	-0.121	0.052	-0.395
Uninsured											1	0.136	0.399	-.549*	0.089	-0.129	-0.081	0.377
smoking												1	0.474	-0.443	.551*	.554*	.527*	0.484
Poverty													1	-.899**	0.235	-0.006	0.495	.618*
MHHI														1	-0.438	-0.059	-0.465	-.773**
Obesity															1	0.359	0.289	.614*
Unemployment																1	0.488	0.105
Total Mines																	1	0.496
HII																		1

*Correlation is significant at p=0.05 **Correlation is significant at p=0.01 level

Table 3. Pearson Correlation Matrix for Non-Metro Counties

	Pop. Density	Native American Population	Estimated Mine Area	Median AI	min.AI	AER	AAP	PM2.5	red air days	Elevation	Uninsured	smoking	Poverty	MHHI	Obesity	Unemploy ment	Total Mines	HII
Pop.density	1	-0.123	-.552*	.593*	0.332	-0.446	0.042	0.372	0.091	0.396	0.075	-0.012	0.032	-0.028	0.054	-0.101	-0.263	-0.005
Native American		1	0.380	-0.246	-0.386	0.283	0.071	-0.407	-0.096	-0.303	.550*	.923**	.921**	-0.315	0.163	.635*	.873**	0.531
Estimated Mine Area			1	-.688**	-.588*	.535*	0.335	-0.492	-0.320	-0.531	0.265	0.485	0.422	-0.095	-0.019	0.383	.689**	0.442
Median AI				1	.795**	-.715**	-0.423	0.450	0.329	.861**	-0.256	-0.181	-0.215	0.123	0.106	-0.460	-0.478	-0.421
min.AI					1	-0.510	-0.252	.633*	.585*	.603*	-0.311	-0.335	-0.338	0.201	0.130	-.682**	-0.529	-0.452
AER						1	0.454	-0.292	-0.262	-.595*	0.244	0.218	0.324	-0.352	-0.277	0.236	0.437	0.359
AAP							1	-0.171	-0.272	-0.269	0.297	0.110	0.292	-0.082	0.072	-0.018	0.255	0.397
PM2.5								1	0.346	0.280	-0.294	-0.414	-0.376	0.321	0.276	-.595*	-0.509	-0.464
red air days									1	-0.074	-0.139	-0.053	-0.127	0.362	-0.205	-0.312	-0.259	0.084
Elevation										1	-0.342	-0.231	-0.269	0.038	0.127	-0.368	-0.417	-.591*
Uninsured											1	.573*	.739**	-.716**	-0.257	0.431	0.387	.760**
smoking												1	.930**	-0.362	0.007	.700**	.889**	.608*
Poverty													1	-0.488	0.063	.592*	.831**	.661*
MHHI														1	0.409	-0.471	-0.246	-0.367
Obesity															1	-0.216	0.206	-0.360
Unemployment																1	.694**	0.440
Total Mines																	1	0.505
HII																		1

*Correlation is significant at p=0.05 **Correlation is significant at p=0.01 level

3.2. Investigating USAD and census tract data through regression kriging

As data at the USAD level (n=99) and census tract level (n=588) were available for the HII [55] (Figure 5b) and CT $PM_{2.5}$, respectively, it was decided to investigate whether these data could be used to gain further insight into AAP and AER patterns. The HII and CT $PM_{2.5}$ data were aggregated to the county-level by averaging the values for the five nearest neighbor USAD/census tracts (Figure 5a-b not shown for CT $PM_{2.5}$) to allow correlation analysis with AAP and AER data at the county-level. The correlations between HII and AAP and AER visits at the county-level were 0.49 and 0.13, respectively. Given the stronger correlation with AAP than AER visits, the HII data were used to regression krige the AAP data to the USAD level (n=99). The AER visit data correlated better with the CT $PM_{2.5}$ data (Summer 2011 max. r=0.323 and Winter 2011-2014 mean r=-0.347) so the modelled $PM_{2.5}$ data were used to regression krige AER visits. The county-level AAP was used as the dependent variable in regression with the county-level HII data as a single independent variable, then a variogram of the regression residuals was computed and modelled. The regression residuals were ordinary kriged to the USAD level. The regression equation from the county-level was then used to predict AAP ($AAP = 0.040558\alpha + 4.884658$ where α corresponds to HII county-level data) at the USAD level and the kriged residuals were added to these regressed values (Figure 2c). To determine the success of the regression kriging, the RK values were aggregated to the county-level using averaging of the five nearest neighbor values (Figure 2d). These values were then correlated and compared with the original county-level AAP values. The correlation coefficient was r=0.93 and the mean RMSE (Figure 2e) was 0.35%. This and the result that follows for AER visits suggests that the combination of using regression association and spatial association in regression kriging is successful at predicting AAP and AER visits at the USAD level and census tract level. The same procedure was followed with the AER data and the CT $PM_{2.5}$ data. The regression equation used was AER=0.3965β0 - 1.4886β1 + 27.915 where β0 and β1 correspond to Summer2011Max and Winter2011to2014Mean, respectively. Once the RK AER data (Figure 2f) were aggregated to the county-level (Figure 2g), the correlation with the original county-level AER data was r=0.876 and the mean RMSE (Figure 2h) was 2.197.

© 2020 by the authors. Licensee MDPI, Basel, Switzerland. This article is an open access article distributed under the terms and conditions of the Creative Commons Attribution (CC BY) license (http://creativecommons.org/licenses/by/4.0/).
